# New Bioactive Polyketides from the Mangrove-Derived Fungus *Penicillium* sp. SCSIO 41411

**DOI:** 10.3390/md22090384

**Published:** 2024-08-26

**Authors:** Yi Chen, Jian Cai, Ziwei Xia, Chunmei Chen, Yonghong Liu, Lalith Jayasinghe, Xueni Wang, Xuefeng Zhou

**Affiliations:** 1 CAS Key Laboratory of Tropical Marine Bio-Resources and Ecology/Guangdong Key Laboratory of Marine Materia Medica, South China Sea Institute of Oceanology, Chinese Academy of Sciences, Guangzhou 510301, China; chenyi221@mails.ucas.ac.cn (Y.C.); caijian19@mails.ucas.ac.cn (J.C.); chenchunmei18@mails.ucas.ac.cn (C.C.); yonghongliu@scsio.ac.cn (Y.L.); lalith.ja@nifs.ac.lk (L.J.); 2 University of Chinese Academy of Sciences, Beijing 100049, China; 3 Guangxi Zhuang Yao Medicine Center of Engineering and Technology, Institute of Traditional Chinese and Zhuang Yao Ethnic Medicine, Guangxi University of Chinese Medicine, Nanning 530200, China; xiaziwei1824@outlook.com; 4 National Institute of Fundamental Studies, Hantana Road, Kandy 200000, Sri Lanka

**Keywords:** mangrove-derived fungus, *Penicillium* sp., polyketide, cytotoxicity

## Abstract

Three new polyketides, including three ester derivatives (**1**, **3**, and **5**) and a new natural product, which was a benzoquinone derivative, embelin A (**4**), together with nine known ones (**2** and **6**–**13**), were isolated from the mangrove-derived fungus *Penicillium* sp. SCSIO 41411. Their structures were determined by detailed NMR and MS spectroscopic analyses. The X-ray single-crystal diffraction analysis of **4** was described for the first time. Compound **9** displayed obvious inhibition against PDE4 with an inhibitory ratio of 40.78% at 10 μM. Compound **12** showed DPPH radical scavenging activity, with an EC_50_ of 16.21 µg/mL, compared to the positive control (ascorbic acid, EC_50_, 11.22 µg/mL). Furthermore, compound **4** exhibited cytotoxicity against PC-3 and LNCaP with IC_50_ values of 18.69 and 31.62 µM, respectively.

## 1. Introduction

Mangroves are a unique intertidal ecosystem located in tropical and subtropical regions, with nearly 60–70% of the world’s tropical and subtropical coastlines covered by mangroves, widely regarded as one of the most productive ecosystems [1,2]. One of the key populations in this ecosystem is microbial diversity [2]. Fungi and bacteria make up 91% of the microbial biomass in tropical mangroves, which produce abundant marine natural products. As of December 2020, scientists have discovered at least 1387 new structures from mangrove-derived fungi, numerous of which demonstrated a variety of pharmacological activities [1,2,3,4].

*Penicillium* sp., as a representative of marine fungi, generates a wide range of bioactive secondary metabolites, mainly including polyketides, alkaloids, peptides, etc. [5,6,7,8]. Auroglaucin, isolated from an aciduric fungus strain, *Penicillium oxalicum* OUCMDZ-5207, exhibited strong selective inhibition on A549 cells, with an IC_50_ value of 5.67 μM [9]. Aspterric acid, which was generated from the fungus *Penicillium polonicum* H175, displayed a noteworthy hypoglycemic impact comparable to that of the positive drug rosiglitazone (RSG) at 10 μM [10].

**Figure 1:**
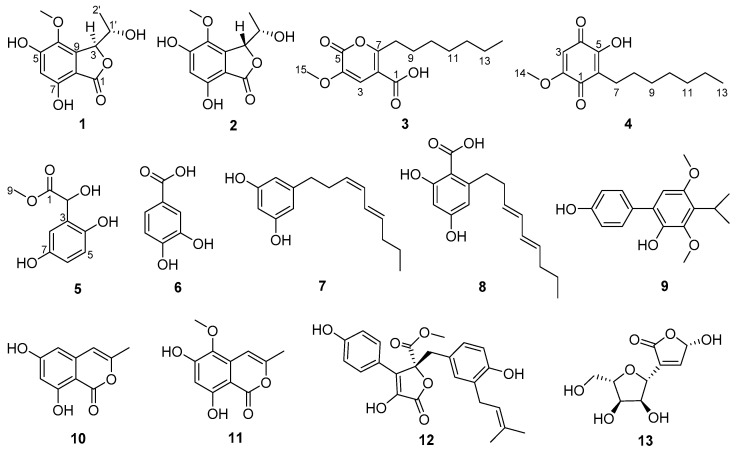
Structures of compounds **1**–**13**.

In our study, three new ester derivatives (**1**, **3**, and **5**) and one new natural product (**4**), together with nine known compounds (**2** and **6**–**13**) (Figure 1), were isolated from a mangrove sediment-derived fungus, *Penicillium* sp. SCSIO 41411. Herein, the specifics of the isolation, structural elucidation, and bioactive assessments of isolated compounds were reported.

## 2. Results and Discussion

### 2.1. Structural Determination

**Figure 2:**
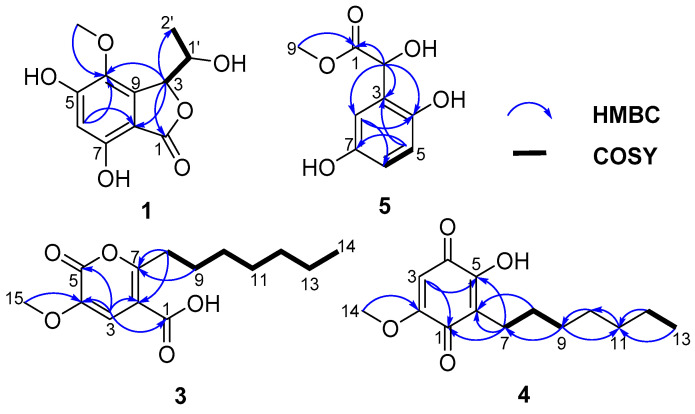
Key HMBC and COSY correlations of **1** and **3**–**5**.

**Figure 3:**
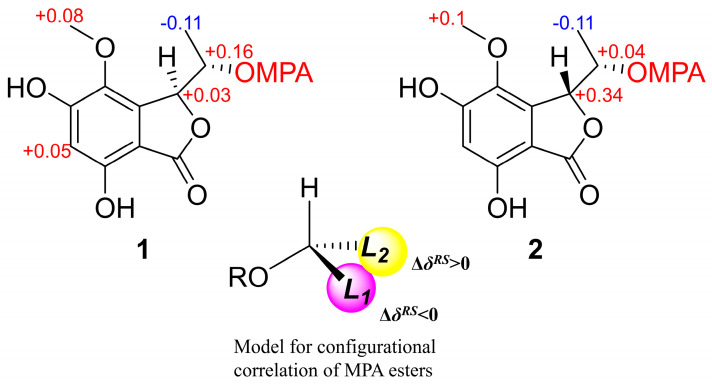
Δ*δ_RS_* (*δ_R_*–*δ_S_*) data for the MPA esters of **1**–**2**.

**Figure 4:**
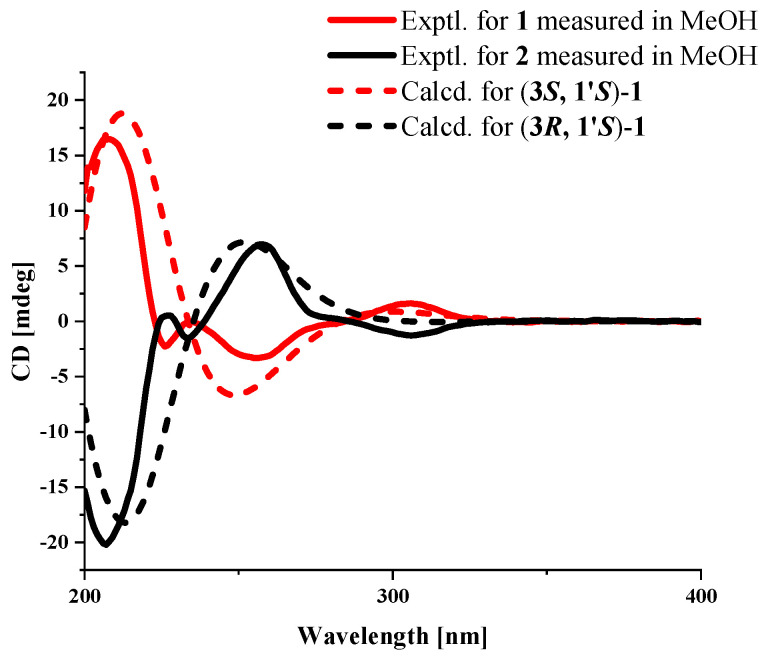
Experimental and calculated ECD spectrum of **1**.

Compound **1** was isolated as a brown oil, and its molecular formula of C_11_H_12_O_6_ was determined by HRESIMS data at *m*/*z* 241.0709 [M+H]^+^. The 1D NMR (Table 1) and HSQC spectra of **1** showed signals of one carbonyl carbon (*δ*_C_ 168.3), five unsaturated carbon signals (*δ*_C_ 157.3, 153.7, 134.3, 141.5, 103.4), one aromatic methine (*δ*_H/C_ 6.41/104.2), two oxygen-containing saturated methylenes (*δ*_H/C_ 5.26/81.5 and 4.18/65.1), and two methyls (*δ*_H/C_ 3.69/59.7 and 1.28/20.6) (one of them was methoxyl). These aforementioned data were similar to the 1D NMR data of the known compound embeurekol C (**2**), a polyketide derivative obtained from the fungus *Embellisia eureka* [11]. HMBC and COSY correlations (Figure 2) confirmed that the planar structures of **1** and **2** were consistent. To determine the absolute configuration of the secondary alcohol at C-1′ in **1** and **2**, we performed the Mosher ester analysis [12]. The (*R*)-MPA and (*S*)-MPA esters were prepared. Analysis of ^1^H NMR data yielded Δ*δ*_RS_ values (*δ*_R_–*δ*_S_), confirming that **1** and **2** had the same *S* configuration at C-1′ (Figure 3). Therefore, two theoretical configurations containing (3*S*, 1′*S*)-**1** and (3*R*, 1′*S*)-**1** were speculated for ECD calculation. Combined with the trend of the experimental curve of **1**, the absolute configuration of **1** was ultimately inferred to be 3*S*, 1′*S* (Figure 4). However, the experimental ECD spectra of **2** showed opposite trends with **1**, so it was speculated that the absolute configuration of C-3 in **2** was opposite to that of **1**, which was 3*R*. The specific optical rotation of **1** (${\left[\alpha \right]}_{D}^{25}$ + 49.0 (*c* 0.1, CH_3_OH)) and embeurekol C (**2**) (${\left[\alpha \right]}_{20_{D}}$ − 17.0 (*c* 0.05, CH_3_OH)) [11] further confirmed their opposite configurations at C-3. Consequently, compound **2** was identical to embeurekol C, and **1** was named embeurekol D.

Compound **3** was obtained as a red-brown solid. The molecular formula was determined as C_14_H_20_O_5_ by HRESIMS data at *m*/*z* 269.1389 [M+H]^+^. The one dimensional (1D) NMR (Table 2) and HSQC spectrum of **3** showed signals of two carbonyls (*δ*_C_ 172.7, 150.2), three nonprotonated sp^2^ carbons (*δ*_C_ 160.0, 153.0, 144.2), one alkene methine (*δ*_H/C_ 6.96/115.6), six methylenes (*δ*_H/C_ 1.24/31.1, 1.30/28.4, 1.30/28.3, 2.68/27.5, 1.61/26.1, 1.24/22.0), and two methyls (*δ*_H/C_ 3.87/53.3, 0.85/13.9) (one of them was methoxyl). According to the HMBC correlation (Figure 2) of H-3/C-1, C-2, C-4, and C-5, compound **3** was an unsaturated pentolactone derivative with a carboxyl group connected to C-2. The HMBC correlations of H_3_-15/C-4 revealed that 15-OCH_3_ was located at C-4. According to the molecular formula, the COSY-related signal indicated a spin-coupled system H_3_-14/H_2_-13/H_2_-12/H_2_-11/H_2_-10/H_2_-9/H_2_-8, indicating the presence of heptane. The HMBC correlations of H_2_-8/C-7, 2, and H_2_-9/C-7 revealed that CH_2_-8 was located at C-7. Compound **3** was unambiguously characterized, as shown in Figure 1. The structure of **3** was similar to that of dothydeopyron B [13], with the main difference being that **3** had a carboxyl group while dothydeopyron B had a methoxy group, and there was a hydroxyl group substituted at the branch. As a newly discovered natural product, the novelty of compound **3** lay in its presence of an aliphatic chain, which made it potentially more hydrophilic compared to compounds with longer chains. Finally, compound **3** was named 7-heptyl-4-methoxy-6-oxo-3*H*-pyran-2-carboxylic acid.

**Figure 5:**
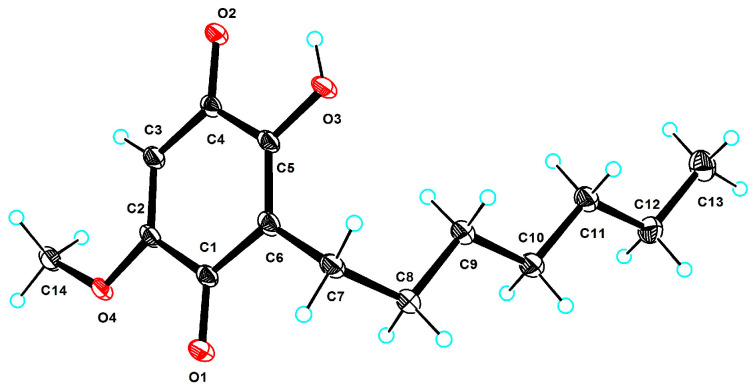
X-ray single-crystal structure of **4**.

Compound **4** was obtained as a yellow solid and was determined to have the molecular formula C_14_H_20_O_4_ from the HRESIMS data at *m*/*z* 253.1442 [M+H]^+^. The 1D NMR (Table 2) and HSQC spectra of **4** showed signals of two carbonyl carbons (*δ*_C_ 184.5, 183.6), three alkene carbons (*δ*_C_ 161.9, 156.1, 120.1), one alkene methine (*δ*_H/C_ 5.88/104.0), six methylenes carbons (*δ*_H/C_ 1.29/33.0, 1.29/30.6, 1.32/30.2, 1.43/29.2, 1.32/23.7, 2.40/23.3), and two methyls (*δ*_H/C_ 3.83/57.2, 0.90/14.4) (one of them was methoxyl). The above data suggested that **4** had a benzoquinone skeleton and a fatty acid chain. The COSY correlations (Figure 2) of H_2_-9/H_2_-8/H_2_-7 and H_2_-12/H_3_-13 and the HMBC correlations of H_3_-13/CH_2_-11 revealed that C-7–C-13 was a fatty acid chain. The HMBC correlations of H_2_-7/C-1, C-5, C-6, and H_2_-8/C-6 revealed that CH_2_-7 was located at C-6. The HMBC correlations of H_3_-14/C-2 indicated that the 14-OCH_3_ was located at C-2. Furthermore, the X-ray crystal structure of **4** (CCDC 2363995, Figure 5) obtained by slow evaporation in CH_3_OH at room temperature further confirmed the above elucidation of the planar structure. Compound **4** has been previously identified as a synthetic product, which was synthesized to illustrate the structure-activity relationship against *α*-glucosidase of the embelin derivatives [14]. Here we reported for the first time that this compound has been explored from nature and confirmed as a new natural product. Therefore, the compound was named embelin A.

Compound **5** was isolated as a brown oil and had the molecular formula C_9_H_10_O_5_ as determined by HRESIMS data at *m*/*z* 221.0429 [M+Na]^+^. The ^1^H NMR of compound **5** shows a typical 1,2,4-trisubstituted benzene ring [*δ*_H_ 6.67 (1H, d, *J* = 3.0 Hz); 6.50 (1H, dd, *J* = 8.5, 3.0 Hz); 6.60 (1H, d, *J* = 8.5 Hz)]. The one-dimensional (1D) NMR (Table 1) and HSQC spectra of 5 showed signals of one carbonyl carbon (*δ*_C_ 173.3), three aromatic carbons (*δ*_C_ 149.7, 146.9, 126.6), three aromatic methines (*δ*_H/C_ 6.60/115.8, 6.50/115.2, and 6.67/114.2), one methine connected to hydroxyl group (*δ*_H/C_ 5.27/67.0), and one oxygenated methyl (*δ*_H/C_ 3.57/51.5). The HMBC correlations (Figure 2) of H_3_-9/C-1 revealed that the oxygenated methyl group 9-OCH_3_ was located at C-1. The HMBC correlations of H-2/C-1, 3, 4, and 8 indicated that C-2 was next to C-1 and C-3. Moreover, it indicated the location of the phenolic hydroxyl group (C-4) and the aromatic methine (C-8). The HMBC correlations of H-8/C-4, 6, and H-5/C-3, 7, and the COSY correlations of H-5/H-6 revealed the location of C-5, C-6, and C-7. The optical rotation of **5** was almost zero, suggesting that it was a racemate. Finally, compound **5** was named methyl *α*,2,5-trihydroxybenzeneacetate.

Meanwhile, the other eight known compounds were identified as protocatechuic acid (**6**) [15], 5-[(3*Z*,5*E*)-3,5-nonadienyl]-1,3-benzenediol (**7**) [16], 2,4-Dihydroxy-6-(3*E*,5*E*)-3,5-nonadien-1-ylbenzoic acid (**8**) [17,18], 3,5-Dimethoxy-4-(1-methylethyl)[1,1′-biphenyl]-2,4′-diol (**9**) [19], 3-Methyl-6,8-dihydroxyisocoumarin (**10**) [20], 6,8-dihydroxy-5-methoxy-3-methyl-1*H*-isochromen-1-one (**11**) [21], butyrolactone I (**12**) [22], polybotrin (**13**) [23], respectively, by comparing their NMR data (Supplementary Information) to previous reports. According to the literature, **11** exhibited *a*-glucosidase inhibitory activity with an IC_50_ value of 89.4 μM [24], and **12** had an acetylcholinesterase inhibiting effect [25].

As we can see, compounds **1/2** and **10/11** had similar parent nucleus structures, with the main difference being that **1/2** were five-membered lactone rings, while compounds **10/11** were six-membered lactone rings. The example of the development of furan and pyran rings in monoterpenoids illustrated the mechanism of biosynthesis. *Aspergillus niger* could be used to biotransformation linalool into both furanoid and pyranoid linalool oxide simultaneously [26]. The phenomena suggested that these two types of compounds had a certain biological relationship in the isolated strain.

### 2.2. Bioactivity Assay

The isolated compounds were evaluated for their antibacterial and cytotoxic activities (Table 3). Compounds **7** and **9** exhibited activities against *Staphylococcus aureus*, with the MIC values of 100 µg/mL. Compounds **3**, **7**, **8**, and **9** exhibited activities against *Streptococcus suis*, with MIC values of 100 µg/mL. In addition, compound **12** had a DPPH inhibitory activity with an EC_50_ of 16.21 µg/mL, compared to the positive control (ascorbic acid, with an EC_50_ of 11.22 µg/mL). Phosphodiesterase 4 (PDE4) is an important target for the treatment of inflammation [27]. In screening for PDE4 inhibitory activity, compounds **1**–**3** and **9**–**13** at 10 µM displayed inhibition against PDE4 with inhibitory ratios of 18.62%, 14.95%, 19.67%, 40.78%, 27.42%, 27.39%, 29.10%, and 26.22%, respectively. Among these, compound **9** exhibited moderate inhibitory activity against PDE4.

**Figure 6:**
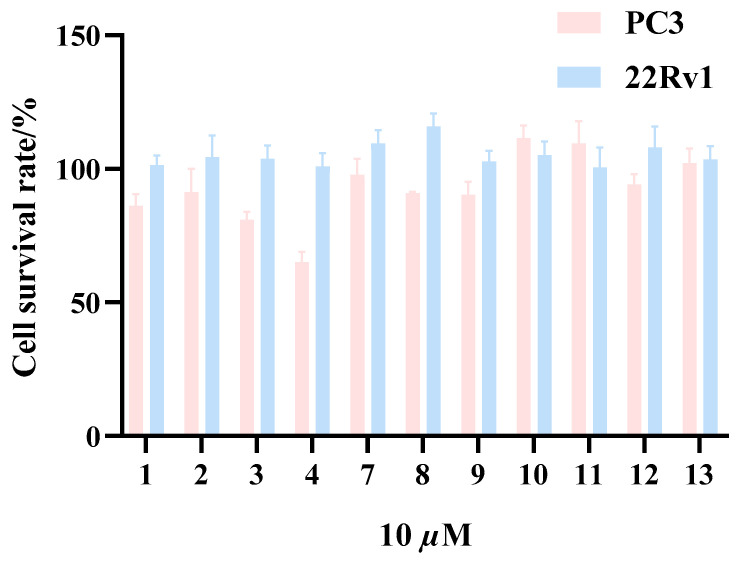
The cell survival rate of compounds **1**–**4** and **7**–**13** at 10 µM. All experiments were performed at least three times. The data are presented as the mean ± SD of representative experiments.

Compounds **1**–**4** and **7**–**13** were tested at 10 µM in the cytotoxicity assay on two human prostate cancer cell lines, PC-3 and 22Rv1 (Figure 6). Compound **4** exhibited significant inhibition on PC-3, with an IC_50_ value of 18.69 µM. Furthermore, Compound **4** was evaluated against two additional prostate cancer cell lines, LNCaP and DU145. It effectively inhibited the LNCaP cell line with an IC_50_ value of 31.62 μM, while its IC_50_ value against the DU145 cell line was greater than 100 μM. The above results indicated that the cytotoxicity activity of **4** was selective, with the strongest activity against the PC-3 cell line. Among these three common prostate cancer cell lines, PC-3 was androgen-independent and had moderate metastatic potential [28]. Therefore, we speculated that compound **4** inhibited the mid-term stage of prostate cancer cell development. As a new natural product, **4** had a similar skeleton structure to auroglaucin-related analogs [29], both of which had a six-membered ring structure and a long chain. However, compounds **3** and **4** differed mostly in the characteristics of their six-membered rings. The cytotoxicity of **4** indicated that lengthy fatty chains had a minimal impact on the cytotoxicity of this kind of structure.

**Figure 7:**
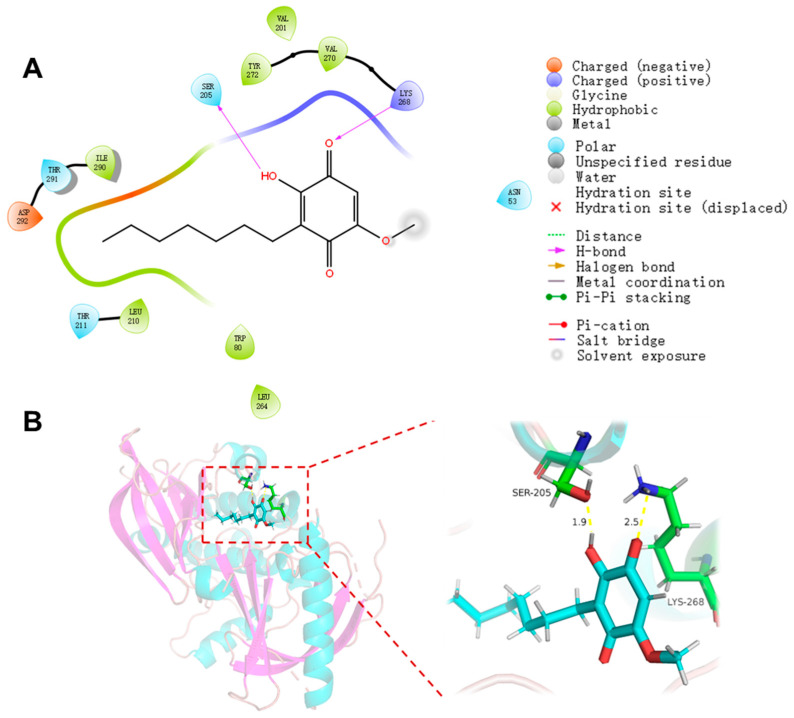
Molecular docking of **4** with AKT1 (PDB code: 3O96). (**A**) The two-dimensional (2D) interaction details of the predicted binding mode of **4** with the AKT1. The purple arrows in the Figure represent hydrogen bonds, indicating that **4** interacted with the active sites of the AKT1 protein pocket through hydrogen bonds. (**B**) The binding sites of molecule **4** with the AKT1 protein. On the left is the overall diagram of the interaction between **4** and AKT1, and on the right is the specific detail diagram. Among them, the distance between **4** and SER 205 is 1.9 Å, and the distance between **4** and LYS 268 is 2.5 Å.

AKT1 (NP_005154.2) was involved in the growth, survival, and metabolism of cells [30]. Studies have shown that AKT1 protein could interact with UHRF1, but inhibited AKT1-induced phosphorylation of UHRF1 could induce degradation of UHRF1 protein, thereby reducing the interaction between UHRF1 and deubiquitinase USP7 and promoting its interaction with E3 ubiquitin-protein ligase BTRC, which is of great significance for the treatment of prostate cancer [31]. Therefore, AKT1 was selected as the docking target for further research. Compound **4** demonstrated a perfect interaction with the AKT1 protein (PDB ID: 3O96) with a docking score of −5.886. As shown in Figure 7, hydroxyl and ketone carbonyl groups of **4** formed two hydrogen bonds with the active site residues SER 205 and LYS 268.

## 3. Materials and Methods

### 3.1. General Experimental Procedures

Optical rotations were measured with an Anton Paar MPC500 (Anton, Graz, Austria) polarimeter. The UV spectra were recorded on a Shimadzu UV-2600 PC spectrometer (Shimadzu, Beijing, China), while the IR spectra were determined by an IR Affinity-1 spectrometer (Shimadzu). The ECD spectra were performed on a Chirascan circular dichroism spectrometer (Applied Photophysics, Leatherhead Surrey, UK). High-resolution electrospray ionization mass spectroscopy (HRESIMS) spectra were obtained on a Bruker maXis Q-TOF mass spectrometer (Bruker BioSpin International AG, Fällanden, Switzerland). The NMR spectra were collected on a Quantum-I Plus 500 MHz (Q-one Instrument Co., Ltd., Wuhan, China) operating at 500 MHz for ^1^H NMR and 125 MHz for ^13^C NMR and were collected on an AVANCE III HD 700 MHz (Bruker Switzerland AG, Fällanden, Switzerland) operating at 700 MHz for ^1^H NMR and 175 MHz for ^13^C NMR, which used tetramethylsilane as an internal standard. Semipreparative high-performance liquid chromatography (HPLC) was performed on the Hitachi Primaide with a DAD detector (Hitachi, Tokyo, Japan), using ODS columns (ChromCore 120 C18, 10 × 250 mm, 5 mm; YMC-pack ODS-A, 10 × 250 mm, 5 mm; COSMOSIL πNAP 10 × 250 mm; COSMOSIL 5C18-AR-II 10 × 250 mm). Column chromatography was detected by silica gel (200–300 mesh), and spots were detected on TLC (Qingdao Marine Chemical Factory, Qingdao, China) under 254 nm UV light, respectively. All solvents used were provided by Tianjin Fuyu Chemical and Industry Factory, Tianjin, China, and were of analytical grade.

### 3.2. Fungal Material

The strain *Penicillium* sp. SCSIO 41411 was isolated from the rhizosphere sediment sample of the mangrove *Aegiceras corniculatum* in Gaoqiao Mangrove, Zhanjiang. It was stored in the CAS Key Laboratory of Tropical Marine Bioresources and Ecology, South China Sea Institute of Oceanology, Chinese Academy of Sciences, Guangzhou, China. The strain was designated *Penicillium* sp. SCSIO 41411 is based on BLAST analysis of the ITS sequence (Supplementary Information) because it shared 99% of the similarities with *Penicillium brefeldianum* (NR_138263.1). Finally, the sequence was deposited in GenBank with the accession number OQ052995.

### 3.3. Fermentation and Extraction

The fungal strain *Penicillium* sp. SCSIO 41411 was statically cultivated on MA medium and then was cultured in 200 mL seed medium (1.5% malt extract, 2.0% sea salt) in 1 L Erlenmeyer flasks at 28 °C for 3 days on a rotary shaker (180 rpm). A large-scale fermentation was incubated at 26 °C for 28 days using a rice medium (200 g rice, 2% sea salt, 230 mL H_2_O) in the 1 L flask (×80) under static conditions. The whole fermented culture was extracted with EtOAc three times to afford a brown extract (150 g).

### 3.4. Isolation and Purification

The whole ethyl acetate extract was subjected to a silica gel vacuum liquid chromatography using a step gradient elution of petroleum ether (PE)-dichloromethane (DCM) (*ν*:*ν* 1:0, 1:1, 0:1), DCM-methyl alcohol (CH_3_OH) (*ν*:*ν* 100:1, 100:3, 50:3, 10:1, 5:1, 10:3, 5:2, 2:1, 10:7, 5:4, 10:9, 0:1), to yield 15 fractions (Frs. 1–15) in the light of TLC profiles. Fr. 2 was further purified by semipreparative HPLC (72% CH_3_OH/H_2_O, 3.0 mL/min) to afford 4 (2.4 mg, *t*_R_ 18.2 min). Fr. 4 to Fr. 6 were merged and then was divided into 18 subfractions (Frs. 4-1–4-18) by ODS silica gel eluting with CH_3_OH/H_2_O (5–100%). Based on this, Fr. 4–8 was directly separated by semipreparative HPLC (69% CH_3_OH/H_2_O, 3.0 mL/min) to offer 12 (37.4 mg, *t*_R_ 21.0 min). Compound **9** (6.3 mg, *t*_R_ 13.3 min) was further purified from Fr. 4–9 by semipreparative HPLC (60% CH_3_CN/H_2_O, 3.0 mL/min). Compound **3** (9.6 mg, *t*_R_ 13.3 min), compound **7** (25.0 mg, *t*_R_ 15.2 min), and Fr. 4-10-1 were further obtained from Fr. 4–10 by semipreparative HPLC (80% CH_3_OH/H_2_O, 2.0 mL/min; 60% CH_3_CN/H_2_O, 2.5 mL/min), respectively. Meanwhile, Fr. 4-10-1 was separated by semipreparative HPLC (52% CH_3_CN/H_2_O, 0.04% formic acid, 3.0 mL/min) to gain 8 (9.7 mg, *t*_R_ 22.0 min). Fr. 4–2 to Fr. 4–7 were merged once again and then were divided into 15 subfractions (Frs. 4-2-1–4-2-15) by ODS silica gel eluting with CH_3_OH/H_2_O (5–100%). Compound **10** (2.3 mg, *t*_R_ 9.8 min) and 11 (12.7 mg, *t*_R_ 10.4 min) were further obtained from Fr. 4-2-5 by semipreparative HPLC (63% CH_3_OH/H_2_O, 2.5 mL/min), respectively. Compound **1** (6.83 mg, *t*_R_ 14.0 min), 5 (2.2 mg, *t*_R_ 9.7 min), 6 (4.5 mg, *t*_R_ 7.8 min) and Fr. 7-5 were firstly obtained from Fr. 7 by semipreparative HPLC (30% CH_3_OH/H_2_O, 2.5 mL/min; 5% CH_3_OH/H_2_O, 0.04% formic acid, 3.0 mL/min; 13% CH_3_CN/H_2_O, 0.04% formic acid, 3.0 mL/min, respectively. Meanwhile, Fr. 7-5 was further separated by semipreparative HPLC (12% CH_3_CN/H_2_O, 0.04% formic acid, 2.5 mL/min) to gain 2 (56.09 mg, *t*_R_ 12.0 min). Compound **13** (24.9 mg, *t*_R_ 7.4 min) was further purified from Fr. 9 by semipreparative HPLC (15% CH_3_OH/H_2_O, 2.5 mL/min).

### 3.5. Spectroscopic Data of Compounds

Embeurekol D (1): brown oil; ${\left[\alpha \right]}_{D}^{25}$ + 49.0 (*c* 0.1, CH_3_OH); UV (CH_3_OH) *λ*_max_ (log*ε*) 214 (4.43), 257 (4.02), 302 (3.85) nm; ECD (0.83 mM, CH_3_OH) *λ*_max_ 208 (+16.47), 226 (−2.28), 234 (−0.02), 256 (−3.33), 306 (+1.62); IR (film) *ν*_max_ 3370, 1732, 1616, 984 cm^−1^; ^1^H and ^13^C NMR data, see Table 1; HRESIMS *m*/*z* 241.0709 [M+H]^+^ (calculated for C_11_H_13_O_6_^+^, 241.0707).

7-heptyl-4-methoxy-6-oxo-3*H*-pyran-2-carboxylic acid (3): red brown solid; UV (CH_3_OH) *λ*_max_ (log*ε*) 226 (4.29), 312 (3.61) nm; IR (film) *ν*_max_ 3256, 2930, 1742, 1636, 1260, 1207 cm^−1^; ^1^H and ^13^C NMR data, see Table 2; HRESIMS *m*/*z* 269.1389 [M+H]^+^ (calculated for C_14_H_21_O_5_^+^, 269.1384).

Embelin A (4): yellow solid; UV (CH_3_OH) *λ*_max_ (log*ε*) 205 (4.05), 287 (4.27) nm; IR (film) *ν*_max_ 3339, 2924, 2853, 1663, 1634, 1593, 1204, 691 cm^−1^; ^1^H and ^13^C NMR data, see Table 2; HRESIMS *m*/*z* 253.1442 [M+H]^+^ (calculated for C_14_H_21_O_4_^+^, 253.1434).

Methyl *α*,2,5-trihydroxybenzeneacetate (5): brown oil; [*α*${]}_{D}^{25}$ 0.0 (*c* 0.1, CH_3_OH); UV (CH_3_OH) *λ*_max_ (log*ε*) 205 (4.16), 301 (3.45) nm; IR (film) *ν*_max_ 3377, 2918, 2851, 1734, 1576, 1456, 1219, 762 cm^−1^; ^1^H and ^13^C NMR data, see Table 1; HRESIMS *m*/*z* 221.0429 [M+Na]^+^ (calculated for C_9_H_10_NaO_5_^+^, 221.0420).

### 3.6. X-ray Crystallographic Analysis

Compound **4** was dissolved in CH_3_OH and slowly evaporated to obtain the clear light crystal. Crystallographic data for the structure that have been submitted to the Cambridge Crystallographic Data Centre (CCDC) can be seen and copies of which can be freely accessed applying to CCDC, 12 Union Road, Cambridge, CB2 1EZ, UK [P: +44 (0) 1223 336408].

Crystal data for 4: C_14_H_20_O_4_, *M*r = 252.30, crystal size 0.3 × 0.05 × 0.05 mm^3^, triclinic, *a* = 5.1888 (8) Å, *b* = 9.6297 (8) Å, *c* = 14.1355 (17) Å, *α* = 74.175 (9)°, *β* = 89.844 (12)°, *γ* = 79.330 (10)°, *V* = 666.93 (15) Å3, *Z* = 2, *T* = 99.98 (10) K, space group *P*-1, *µ* (Cu Kα) = 0.746 mm^−1^, *D*_calc_ = 1.256 g/cm^3^, 4793 reflections measured (6.508° ≤ 2*Θ* ≤ 151.438°), 2535 unique (*R*_int_ = 0.0620, *R*_sigma_ = 0.0720). The final *R*_1_ was 0.0950 (*I* > 2*σ*(*I*)), and *wR*_2_ was 0.2899 (all data). The goodness of fit on *F*^2^ was 1.090 (CCDC 2363995).

### 3.7. ECD Computation Section

Compounds **1** and **2** were placed in Spartan’14, which used the MMFF molecular force field to perform a conformational search on the potential isomers separately. Then, the stable conformers of the first 5% in methanol solvent were optimized at the B3LYP/6-31+G (d) level via Gaussian 09 (D.01, Pittsburgh, PA, USA). A TDDFT polarizable continuum model at the level of B3LYP/6-311+G (d, p) was used to calculate the optimized low-energy conformations [32]. The calculated ECD spectra were generated from GaussView (6.0.16, Pittsburgh, PA, USA) and Origin 2021 with a half-bandwidth of 0.3–0.4 eV, wavelength corrected by the calculated UV curve with the measured curve and weighted by Boltzmann distribution to obtain the calculated ECD spectra.

### 3.8. Mosher’s Method

Compound **1** (2.01 mg, 0.0084 mmol), followed by the addition of (*R*)-MPA (8.50 mg, 0.0512 mmol), DCC (0.3455 mg, 0.0017 mmol), and DMAP (0.2046 mg, 0.0017 mmol), was dissolved in chloroform-*d* (0.6 mL) and then stirred at room temperature for 6 h. The reaction was monitored by TLC detection [12]. (*R*)-MPA ester (1a) was purified by semipreparative HPLC (60% CH_3_OH/H_2_O, 3.0 mL/min) to yield 1a (0.7 mg). Compound **1b** (0.7 mg) was prepared using the same protocol as for 1a. The determination of the configuration of the hydroxyl group at C-1′ for 2 was performed the same way as for 1.

### 3.9. Antibacterial Activity Assay

Two bacteria (*Staphylococcus aureus* ATCC 25923, *Streptococcus suis* SC19) were assessed on 96-well plates. As positive controls against the two microorganisms, streptomycin and penicillin were employed, respectively. Then, compounds were assessed using a two-fold serial dilution approach on nutrient agar on a 96-well plate as previously described [33]. The medium’s OD value was determined at 490 nm (OD_490_) using a microplate reader (Thermo Scientific, Bremen, Germany) following a 24-h incubation period.

### 3.10. Antioxidant Activity Assay

The obtained compound **12** was evaluated for its antioxidant activities against DPPH. The effect of the compound on DPPH radical was estimated according to the method of Hatano et al. (1988) [34] and Yen et al. (1995) [35]. In summary, a methanolic DPPH solution was supplemented with compound **12** to obtain a final concentration of 2.5–250 μg/mL. After shaking the mixture and letting it stand for 30 min at room temperature in the dark, the OD_517_ values were measured using the PerkinElmer Enspire Multi-mode micro-orifice detector and enzyme labeling instrument (PerkinElmer, Waltham, MA, USA). Ascorbic acid was used as the positive control. Then, the free radical scavenging rate K (%) was computed based on the acquired OD_517_ value using the formula, and Origin 2021 was utilized to determine EC_50_.

### 3.11. PDE4 Inhibitory Screening Assays

The PDE4D2 expression, purification, and enzymatic assay procedures were comparable to those we previously described [36]. In brief, the inhibition of PDE4D2 by compounds **1**–**3** and **9**–**13** was measured using ^3^H-cAMP as substrates (20,000–30,000 cpm/assay), and reactions took place in a mixture that also contained 50 mM Tris-HCl (pH 7.5), 10 mM MgCl_2_, 0.5 mM (DTT) at room temperature (25°) for 15 min. Subsequently, the reaction was stopped with 0.2 M ZnSO_4_, the unreacted ^3^H-cAMP was precipitated out using 0.2 N Ba(OH)_2_. The leftover supernatant was used to gauge the radioactivity in 2.5 mL of Ultima Gold liquid scintillation cocktails (PerkinElmer) by a liquid scintillation counter (PerkinElmer 2910) [36].

### 3.12. Cytotoxicity Bioassay

Human prostate cancer LNCaP, 22Rv1, PC-3, and DU145 cells were purchased from the Cell Bank/Stem Cell Bank of the Chinese Academy of Sciences (Shanghai, China). All cell lines were cultured in the medium according to the recommendations of the Chinese Academy of Sciences Cell Bank/Stem Cell Bank. The cells were incubated at 37 °C and 5% carbon dioxide. All culture media supplemented with 10% (*v*/*v*) fetal bovine serum (Biological Industries, Beit Haemek, Israel) and 1% penicillin/streptomycin. All cell lines were tested and found to be free of mycoplasma contamination. The MTT assay, which has been previously reported, was used to assess cytotoxicity [37]. To sum up, cells were treated with compounds after being seeded at a density of 5 × 10^3^ per well on a 96-well plate overnight after 72 h incubation. Following drug treatment, 10 μL of MTT solution (5 mg/mL) was added to each well at the indicated timings, and the wells were then incubated for 4 h at 37 °C in a humidified 5% CO_2_ incubator. After the supernatants were eliminated, DMSO (100 μL) was added. Then, the OD_570_ values after 10 min were measured using a TECAN Infinite 200 PRO Nano Quant multimode microplate reader [37,38]. Three separate runs of the experiment were conducted.

### 3.13. Molecular Docking

Using the structure of AKT1 (PDB code: 3O96) [30]**,** which was obtained from the Protein Data Bank as a starting model, it was then handled following the Protein Preparation Wizard workflow in Maestro 11.9. The optimized ligand was docked into the receptor with the default parameters, which were obtained from Receptor Grid Generation [39,40,41,42].

## 4. Conclusions

In conclusion, three new polyketides (**1**, **3**, and **5**) and one new natural product (**4**), together with nine known ones (**2** and **6**–**13**), were isolated from the mangrove sediment-derived fungus *Penicillium* sp. SCSIO 41411. Their structures were determined by extensive spectroscopic analyses, ECD calculations, and X-ray single-crystal diffraction. Compound **9** inhibited PDE4 with an inhibitory ratio of 40.78% at 10 μM. Compound **12** showed moderate antioxidant inhibitory activity with an EC_50_ of 16.21 µg/mL. Moreover, compound **4** demonstrated selective inhibitory activity against tumor cells, which provides a promising avenue for the development of anti-prostate cancer therapeutics.

## Figures and Tables

**Table 1:** The ^1^H and ^13^C NMR data of compounds **1** and **5**.

Pos.	1 ^a^		5 ^b^	
	*δ*_C_ Type	*δ*_H_ (*J* in Hz)	*δ*_C_ Type	*δ*_H_ (*J* in Hz)
1	168.3, C		173.3, C	
2			67.0, CH	5.27, s
3	81.5, CH	5.26, br s	126.6, C	
4	134.3, C		146.9, C	
5	157.3, C		115.8, CH	6.60, d (8.5)
6	104.2, CH	6.41, s	115.2, CH	6.50, dd (8.5, 3.0)
7	153.7, C		149.7, C	
8	103.4, C		114.2, CH	6.67, d (3.0)
9	141.5, C		51.5, CH_3_	3.57, s
10	59.7, CH_3_	3.69, s		
1′	65.1, CH	4.18, q (5.9)		
2′	20.6, CH_3_	1.28, d (6.5)		

^a 1^H (500 MHz) and ^13^C (125 MHz) measured in DMSO-*d*_6_. ^b 1^H (700 MHz) and ^13^C (175 MHz) measured in DMSO-*d*_6_.

**Table 2:** The ^1^H and ^13^C NMR data of compounds **3** and **4**.

Pos.	3 ^a^		4 ^b^	
	*δ*_C_ Type	*δ*_H_ (*J* in Hz)	*δ*_C_ Type	*δ*_H_ (*J* in Hz)
1	172.7, C		183.6, C	
2	144.2, C		161.9, C	
3	115.6, CH	6.96, s	104.0, CH	5.88, s
4	160.0, C		184.5, C	
5	150.2, C		156.1, C	
6			120.1, C	
7	153.0, C		23.3, CH_2_	2.40, t (7.7)
8	27.5, CH_2_	2.68, t (7.5)	29.2, CH_2_	1.43, m
9	26.1, CH_2_	1.61, m	30.2, CH_2_	1.32, m
10	28.3, CH_2_	1.30, m	30.6, CH_2_	1.29, m
11	28.4, CH_2_	1.30, m	33.0, CH_2_	1.29, m
12	31.1, CH_2_	1.24, m	23.7, CH_2_	1.32, m
13	22.0, CH_2_	1.24, m	14.4, CH_3_	0.90, t (7.1)
14	13.9, CH_3_	0.85, t (6.8)	57.2, CH_3_	3.83, s
15	53.3, CH_3_	3.87, s		

^a 1^H (500 MHz) and ^13^C (125 MHz) measured in DMSO-*d*_6_. ^b 1^H (700 MHz) and ^13^C (175 MHz) measured in CD_3_OD.

**Table 3:** Antibacterial and cytotoxicity activities of compounds.

Compounds	Antibacterial (MIC, µg/mL)		Cytotoxicity (IC_50_, µM)		
	*S. aureus*	*S. suis*	PC-3	LNCaP	DU145
**1**	- ^a^	-	-	-	-
**3**	-	100	-	-	-
**4**	-	-	18.69	31.62	>100
**7**	100	100	-	-	-
**8**	-	100	-	-	-
**9**	100	100	-	-	-
**12**	-	-	-	-	-
**Positive**	25 ^b^	50 ^c^	0.12 ^d^	0.0018 ^d^	0.0033 ^d^

^a^ “-” means no activity; ^b^ Streptomycin; ^c^ Penicillin; ^d^ Docetaxel.

## Data Availability

Data are contained within the article.

## Supplementary Materials

The following supporting information can be downloaded at https://www.mdpi.com/article/10.3390/md22090384/s1, physicochemical data of **2** and **6**–**13**; Figures S1–S55: The NMR, HRESIMS, UV, and IR spectra of **1**–**13**; ITS sequence data of the strain.
