# Supplementary material for: New Bioactive Polyketides from the Mangrove-Derived Fungus Penicillium sp. SCSIO 41411
Source: Mar Drugs. 2024 Aug 26;22(9):384. doi: 10.3390/md22090384 (PMC11433107; doi:10.3390/md22090384)
Supplement: Supplementary file 1 [file marinedrugs-22-00384-s001.zip › marinedrugs-3136919-supplementary.pdf]

Supporting Information

New Bioactive Polyketide Metabolites from the Mangrove-Derived Fungus *Penicillium* sp. SCSIO 41411

Yi Chen [1,2,#], Jian Cai[1,2,#], Ziwei Xia[3], Chunmei Chen[1], Yonghong Liu[1, 3], Lalith Jayasinghe[1, 4], Xueni Wang[3,*], Xuefeng Zhou[1,2,*]

[1] CAS Key Laboratory of Tropical Marine Bio-resources and Ecology/Guangdong Key Laboratory of Marine Materia Medica, South China Sea Institute of Oceanology, Chinese Academy of Sciences, Guangzhou 510301, China;

[2] University of Chinese Academy of Sciences, Beijing 100049, China;

[3] Guangxi Zhuang Yao Medicine Center of Engineering and Technology, Institute of Traditional Chinese and Zhuang-Yao Ethnic Medicine, Guangxi University of Chinese Medicine, Nanning 530200, China.

[4] National Institute of Fundamental Studies, Hantana Road, Kandy, Sri Lanka.

Correspondence: wangxueni@gxtcmu.edu.cn; xfzhou@scsio.ac.cn

# These authors contributed equally to this work.

The physicochemical data of the known compounds 2, 6–13. 4

Figure S1: ^{1}H NMR spectrum of 1 in DMSO-d_6. 6

Figure S2: ^{13}C NMR spectrum of 1 in DMSO-d_6. 6

Figure S3: HSQC spectrum of 1 in DMSO-d_6. 7

Figure S4: HMBC spectrum of 1 in DMSO-d_6. 7

Figure S5: COSY spectrum of 1 in DMSO-d_6. 8

Figure S6: NOESY spectrum of 1 in DMSO-d_6. 8

Figure S7: HRESIMS spectrum of 1. 10

Figure S8: IR spectrum of 1. 11

Figure S9: UV spectrum of 1 in MeOH. 11

Figure S10: ECD spectrum of 1 in MeOH. 12

Figure S11: ^{1}H NMR spectrum of 2 in DMSO-d_6. 13

Figure S12: ^{13}C NMR spectrum of 2 in DMSO-d_6. 13

Figure S13: ^{1}H NMR spectrum of 3 in DMSO-d_6. 14

Figure S14: ^{13}C NMR spectrum of 3 in DMSO-d_6. 14

Figure S15: HSQC spectrum of 3 in DMSO-d_6. 15

Figure S16: HMBC spectrum of 3 in DMSO-d_6. 15

Figure S17: COSY spectrum of 3 in DMSO-d_6. 16

Figure S18: NOESY spectrum of 3 in DMSO-d_6. 16

Figure S19: HRESIMS spectrum of 3. 17

Figure S20: IR spectrum of 3. 18

Figure S21: UV spectrum of 3 in MeOH. 18

Figure S22: ^{1}H NMR spectrum of 4 in Methanol-d_4. 19

Figure S23: ^{13}C NMR spectrum of 4 in Methanol-d_4. 19

Figure S24: HSQC spectrum of 4 in Methanol-d_4. 20

Figure S25: HMBC spectrum of 4 in Methanol-d_4. 20

Figure S26: COSY spectrum of 4 in Methanol-d_4. 21

Figure S27: NOESY spectrum of 4 in Methanol-d_4. 21

Figure S28: HRESIMS spectrum of 4. 22

Figure S29: IR spectrum of 4. .... **23**

Figure S30: UV spectrum of 4 in MeOH. **23**

Figure S31: ^{1}H NMR spectrum of 5 in DMSO-d_6. .... **24**

Figure S32: ^{13}C NMR spectrum of 5 in DMSO-d_6. .... **24**

Figure S33: HSQC spectrum of 5 in DMSO-d_6. .... **25**

Figure S34: HMBC spectrum of 5 in DMSO-d_6. .... **25**

Figure S35: COSY spectrum of 5 in DMSO-d_6. .... **26**

Figure S36: NOESY spectrum of 5 in DMSO-d_6. .... **26**

Figure S37: HRESIMS spectrum of 5. .... **27**

Figure S38: IR spectrum of 5. .... **28**

Figure S39: UV spectrum of 5 in MeOH. **29**

Figure S40: ^{1}H NMR spectrum of 6 in DMSO-d_6. .... **29**

Figure S41: ^{13}C NMR spectrum of 6 in DMSO-d_6. .... **30**

Figure S42: ^{1}H NMR spectrum of 7 in DMSO-d_6. .... **30**

Figure S43: ^{13}C NMR spectrum of 7 in DMSO-d_6. .... **31**

Figure S44: ^{1}H NMR spectrum of 8 in DMSO-d_6. .... **31**

Figure S45: ^{13}C NMR spectrum of 8 in DMSO-d_6. .... **32**

Figure S46: ^{1}H NMR spectrum of 9 in DMSO-d_6. .... **32**

Figure S47: ^{13}C NMR spectrum of 9 in DMSO-d_6. .... **33**

Figure S48: ^{1}H NMR spectrum of 10 in DMSO-d_6. .... **33**

Figure S49: ^{13}C NMR spectrum of 10 in DMSO-d_6. .... **34**

Figure S50: ^{1}H NMR spectrum of 11 in DMSO-d_6. .... **34**

Figure S51: ^{13}C NMR spectrum of 11 in DMSO-d_6. .... **35**

Figure S52: ^{1}H NMR spectrum of 12 in DMSO-d_6. .... **35**

Figure S53: ^{13}C NMR spectrum of 12 in DMSO-d_6. .... **36**

Figure S54: ^{1}H NMR spectrum of 13 in DMSO-d_6. .... **36**

Figure S55: ^{13}C NMR spectrum of 13 in DMSO-d_6. .... **36**

ITS sequence of the strain *Penicillium* sp. SCSIO 41411 .... **37**

The physicochemical data of the known compounds 2, 6–13.

Embeurekol C (2): Brown oil; ^{1}H NMR (500 MHz, DMSO-d_6) δ_H 6.41 (1H, s, H-6), 5.45 (1H, d, J = 2.0 Hz, H-3), 4.25 (1H, m, H-1′), 3.67 (3H, s, H-10), 0.72 (3H, d, J = 6.4 Hz, H-2′); ^{13}C NMR (125 MHz, DMSO-d_6) δ_C 168.8 (C, C-1), 157.6 (C, C-5), 154.2 (C, C-7), 140.8 (C, C-9), 134.8 (C, C-4), 104.9 (CH, C-6), 103.2 (C, C-8), 82.5 (CH, C-3), 66.7 (CH, C-1′), 60.4 (4-OCH_3, C-10), 15.4 (CH_3, C-2′).

Protocatechuic acid (6): Brown oil; ^{1}H NMR (500 MHz, DMSO-d_6) δ_H 8.57 (1H, s), 6.56 (1H, d, J = 8.5 Hz, H-2), 6.51 (1H, d, J = 2.9 Hz, H-5), 6.44 (1H, dd, J = 8.5, 2.9 Hz, H-6); ^{13}C NMR (125 MHz, DMSO-d_6) δ_C 173.1 (C, C-7), 149.4 (C, C-4), 148.0 (C, C-3), 122.6 (C, C-1), 117.4 (CH, C-6), 115.5 (CH, C-5), 113.9 (CH, C-2).

5-[(3*Z*,5*E*)-3,5-nonadienyl]-1,3-benzenediol (7): Brown oil; ^{1}H NMR (500 MHz, DMSO-d_6) δ_H 6.03 (2H, d, J = 2.1 Hz, H-4, 6), 6.02 (1H, d, J = 2.1 Hz, H-2), 5.97 (2H, m, H-10, 11), 5.56 (2H, m, H-9, 12), 2.44 (2H, m, H-7), 2.25 (2H, m, H-8, 13), 1.99 (2H, m, H-8, 13), 1.35 (2H, m, H-14), 0.84 (3H, t, J = 7.4 Hz, H-15); ^{13}C NMR (125 MHz, DMSO-d_6) δ_C 158.2 (2C, C-1, 3), 143.5 (C, C-5), 132.1 (CH, C-12), 131.4 (CH, C-10), 130.6 (CH, C-9), 130.5 (CH, C-11), 106.3 (2CH, C-4, 6), 100.2 (CH, C-2), 35.3 (CH_2, C-7), 34.1 (CH_2, C-13), 33.7 (CH_2, C-8), 22.1 (CH_2, C-14), 13.6 (CH_3, C-15).

2,4-Dihydroxy-6-(3*E*,5*E*)-3,5-nonadien-1-ylbenzoic acid (8): Red brown solid; ^{1}H NMR (500 MHz, DMSO-d_6) δ_H 6.11 (1H, d, J = 2.4 Hz, H-2), 6.09 (1H, d, J = 2.4 Hz, H-4),5.98 (2H, m, H-11, 12), 5.56 (2H, m, H-10, 13), 2.86 (2H, t, J = 7.8 Hz, H-8), 2.24 (2H, q, J = 7.4 Hz, H-9), 2.00 (2H, q, J = 7.1 Hz, H-14), 1.34 (2H, m, H-15), 0.85 (3H, t, J = 7.3 Hz, H-16); ^{13}C NMR (125 MHz, DMSO-d_6) δ_C 172.7 (C, C-7), 164.3 (C, C-1), 161.1 (C, C-3), 158.2 (C, C-6), 146.3 (C, C-5), 132.0 (CH, C-13), 131.8 (CH, C-10), 130.5 (CH, C-11), 130.4 (CH, C-12), 109.6 (CH, C-2), 100.7 (CH, C-4), 35.3 (CH_2, C-8), 34.3 (CH_2, C-9), 34.1 (CH_2, C-14), 22.1 (CH_2, C-15), 13.6 (CH_3, C-16).

3,5-Dimethoxy-4-(1-methylethyl)[1,1′-biphenyl]-2,4′-diol (9): Brown oil; ^{1}H NMR (500 MHz, DMSO-d_6) δ_H 7.37 (2H, m, H-8, 12), 6.78 (2H, m, H-9, 11), 6.56 (1H, s, H-6), 3.72 (3H, s, 5-OCH_3), 3.65 (3H, s, 3-OCH_3), 3.35 (1H, m, H-17), 1.28 (3H, s, H-18/19), 1.27 (3H, s, H-18/19); ^{13}C NMR (125 MHz, DMSO-d_6) δ_C 156.3 (C, C-10), 151.2 (C, C-3), 146.8 (C, C-5), 140.8 (C, C-2), 130.2 (2CH, C-8, 12), 129.2 (C, C-7), 127.4 (C, C-1), 126.5 (C, C-4), 114.8 (2CH, C-9, 11), 108.6 (CH, C-6), 60.9 (CH_3, 3-OCH_3), 55.9 (CH_3, 5-OCH_3), 25.0 (CH, C-17), 21.3 (2CH_3, C-18, 19).

3-Methyl-6,8-dihydroxyisocoumarin (10): Orange crystal; ^{1}H NMR (700 MHz, DMSO-d_6) δ_H 6.43 (1H, s, H-4), 6.31 (1H, s, H-5), 6.28 (1H, s, H-7), 2.18 (3H, s, H-9); ^{13}C NMR (175 MHz, DMSO-d_6) δ_C 166.3 (C, C-6), 165.7 (C, C-1), 162.9 (C, C-8), 154.4 (C, C-3), 139.9 (C, C-4a), 104.4 (CH, C-4), 102.8 (CH, C-5), 101.7 (CH, C-7), 97.9 (C, C-8a), 19.1 (CH_3, C-9).

6,8-dihydroxy-5-methoxy-3-methyl-1*H*-isochromen-1-one (11): Orange crystal; ^{1}H NMR (500 MHz, DMSO-d_6) δ_H 10.74 (1H, s), 6.59 (1H, brs, H-7), 6.43 (1H, s, H-4), 3.69 (3H, s, 5-OCH_3), 2.24 (3H, brs, H-9); ^{13}C NMR (125 MHz, DMSO-d_6) δ_C 165.4 (C, C-1), 158.7 (C, C-6), 158.5 (C, C-8), 154.1 (C, C-3), 133.6 (C, C-4a), 131.0 (C, C-5), 102.1 (CH, C-7), 98.7 (C, C-8a), 96.8 (CH,

C-4), 60.8 (CH_3, 5-OCH_3), 19.1 (CH_3, C-9).

Butyrolactone I (12): Brown oil; ^1H NMR (500 MHz, DMSO-d_6) δ_H 10.01 (1H, s), 9.21 (1H, s), 7.49 (2H, d, J = 8.6 Hz, H-2′, 6′), 6.87 (2H, d, J = 8.7 Hz, H-3′, 5′), 6.52 (1H, d, J = 8.2 Hz, H-5″), 6.47 (1H, dd, J = 8.2, 2.2 Hz, H-6″), 6.36 (1H, d, J = 2.2 Hz, H-2″), 4.99 (1H, m, H-8″), 3.73 (3H, s, 5-OCH_3), 3.36 (2H, s, H-6), 2.99 (2H, t, J = 6.4 Hz, H-7″), 1.61 (3H, s, H-10″/11″), 1.52 (3H, s, H-10″/11″); ^{13}C NMR (125 MHz, DMSO-d_6) δ_C 170.0 (C, C-5), 168.1 (C, C-1), 158.0 (C, C-4′), 153.9 (C, C-4″), 138.2 (C, C-2), 131.6 (C, C-9″), 131.0 (CH, C-2″), 128.9 (2CH, C-2′, 6′), 128.5 (CH, C-6″), 127.5 (C, C-3″), 126.6 (C, C-3), 123.3 (C, C-1″), 122.4 (CH, C-8″), 121.2 (C, C-1′), 115.9 (2CH, C-3′, 5′), 114.2 (CH, C-5″), 84.9 (C, C-4), 53.6 (CH_3, 5-OCH_3), 38.2 (CH_2, C-6), 27.6 (CH_2, C-7″), 25.6 (CH_3, C-10″/11″), 17.6 (CH_3, C-10″/11″).

Polybotrin (13): Brown oil; ^1H NMR (500 MHz, DMSO-d_6) δ_H 7.88 (1H, d, J = 8.1 Hz, H-4′), 5.78 (1H, d, J = 5.4 Hz, H-2), 5.64 (1H, d, J = 8.1 Hz, H-5′), 4.02 (1H, t, J = 5.3 Hz, H-3), 3.96 (1H, t, J = 4.6 Hz, H-4), 3.84 (1H, q, J = 3.4 Hz, H-5), 3.62 (1H, dd, J = 11.9, 3.2 Hz, H-6), 3.54 (1H, dd, J = 12.0, 3.3 Hz, H-6); ^{13}C NMR (125 MHz, DMSO-d_6) δ_C 163.1 (C, C-2′), 150.8 (C, C-3′), 140.7 (CH, C-4′), 101.8 (CH, C-5′), 87.7 (CH, C-2), 84.8 (CH, C-5), 73.5 (CH, C-3), 69.9 (CH, C-4), 60.9 (CH_2, C-6).

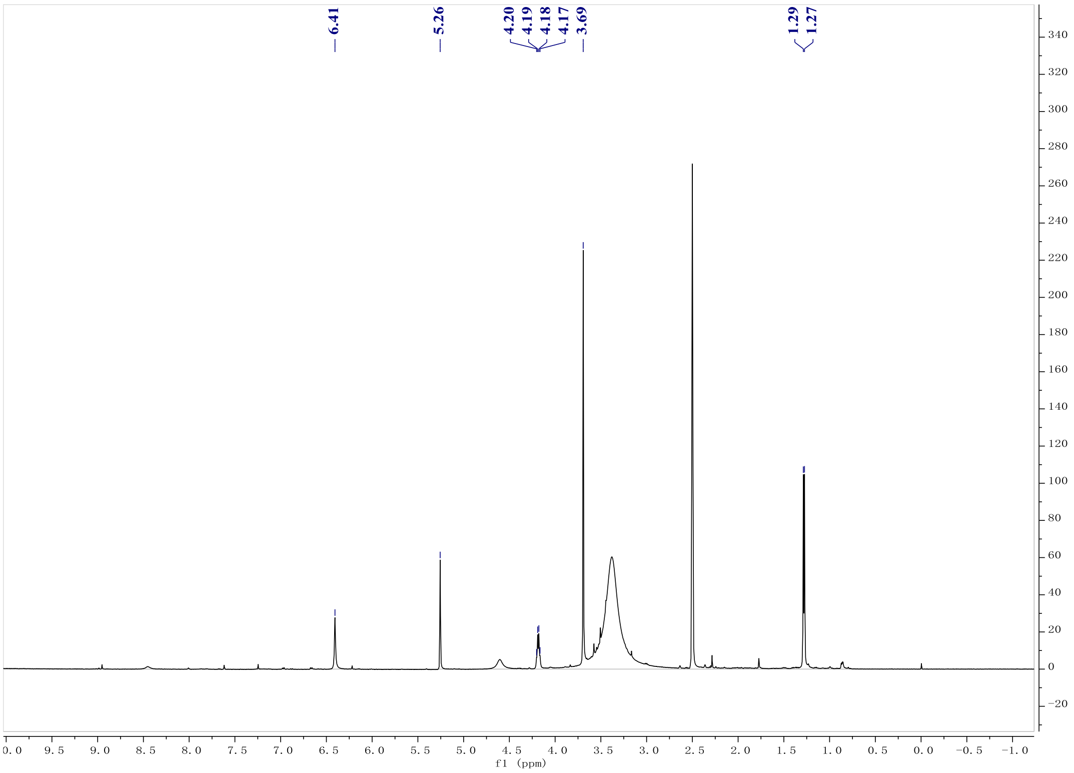

Figure S1: ^{1}H NMR spectrum of **1** in DMSO-d_6.

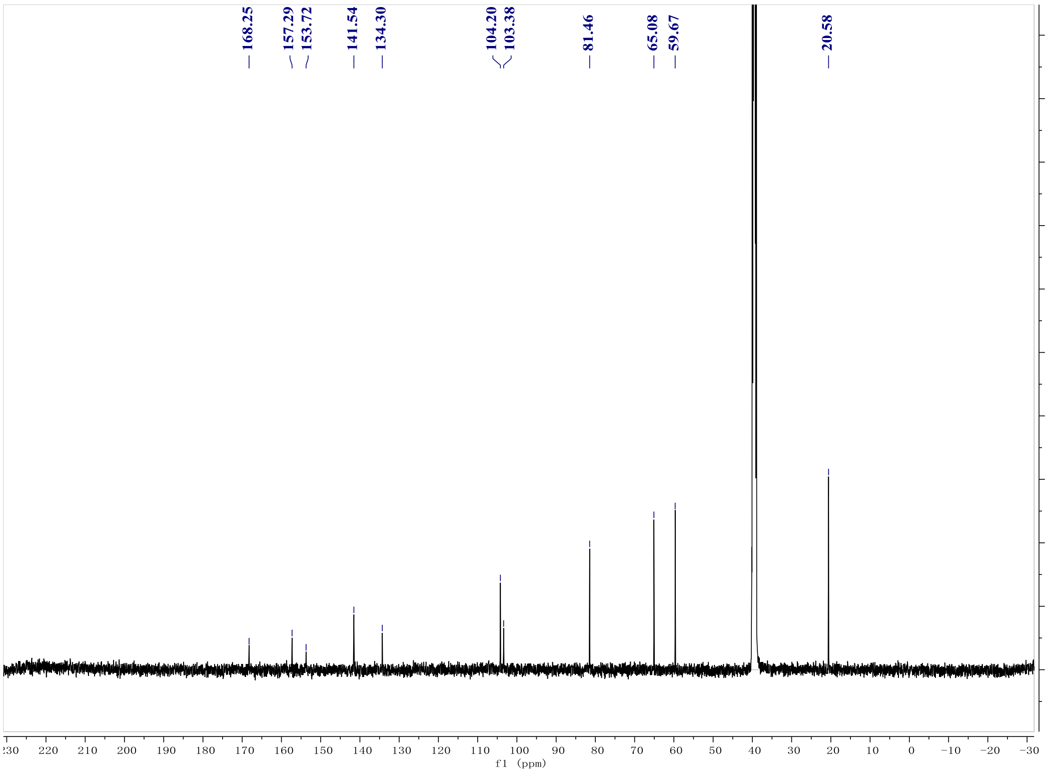

Figure S2: ^{13}C NMR spectrum of **1** in DMSO-d_6.

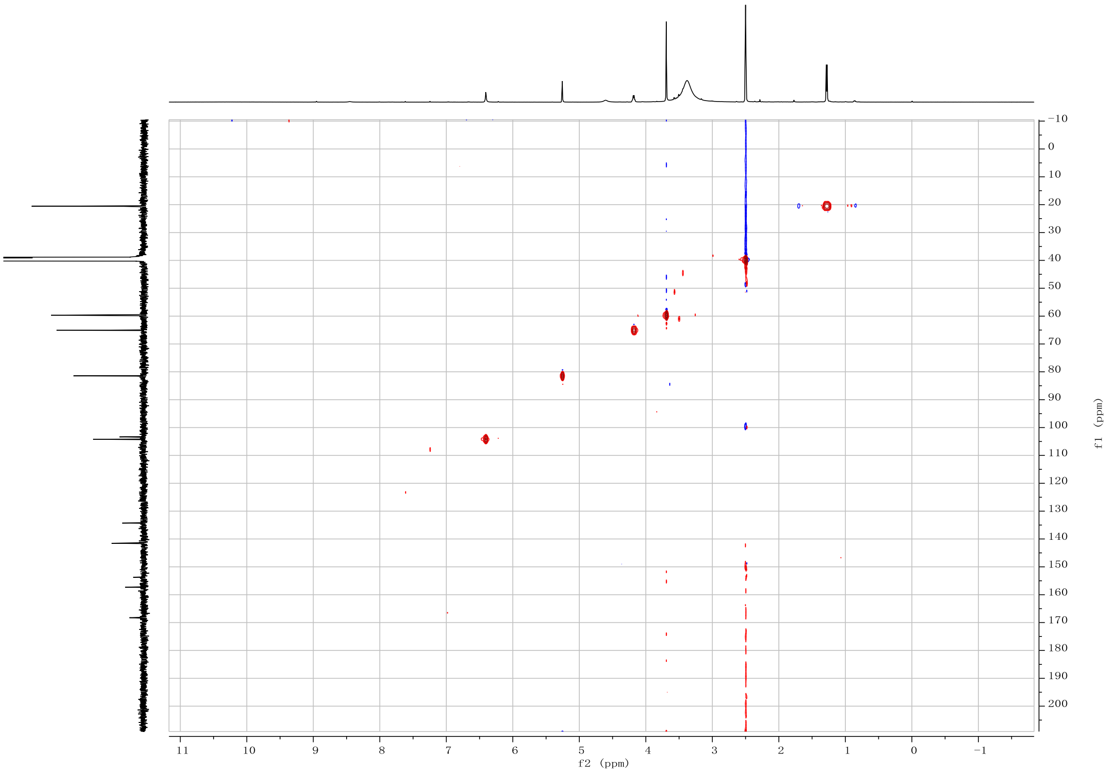

Figure S3: HSQC spectrum of **1** in DMSO-d_6.

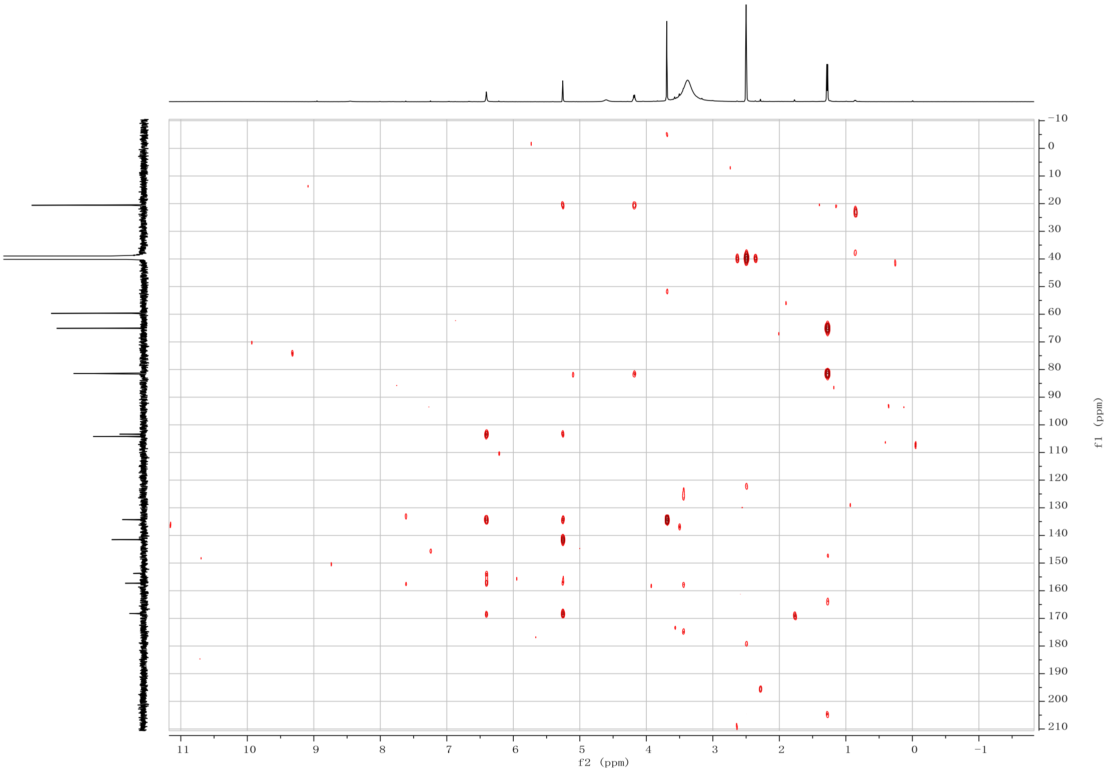

Figure S4: HMBC spectrum of **1** in DMSO-d_6.

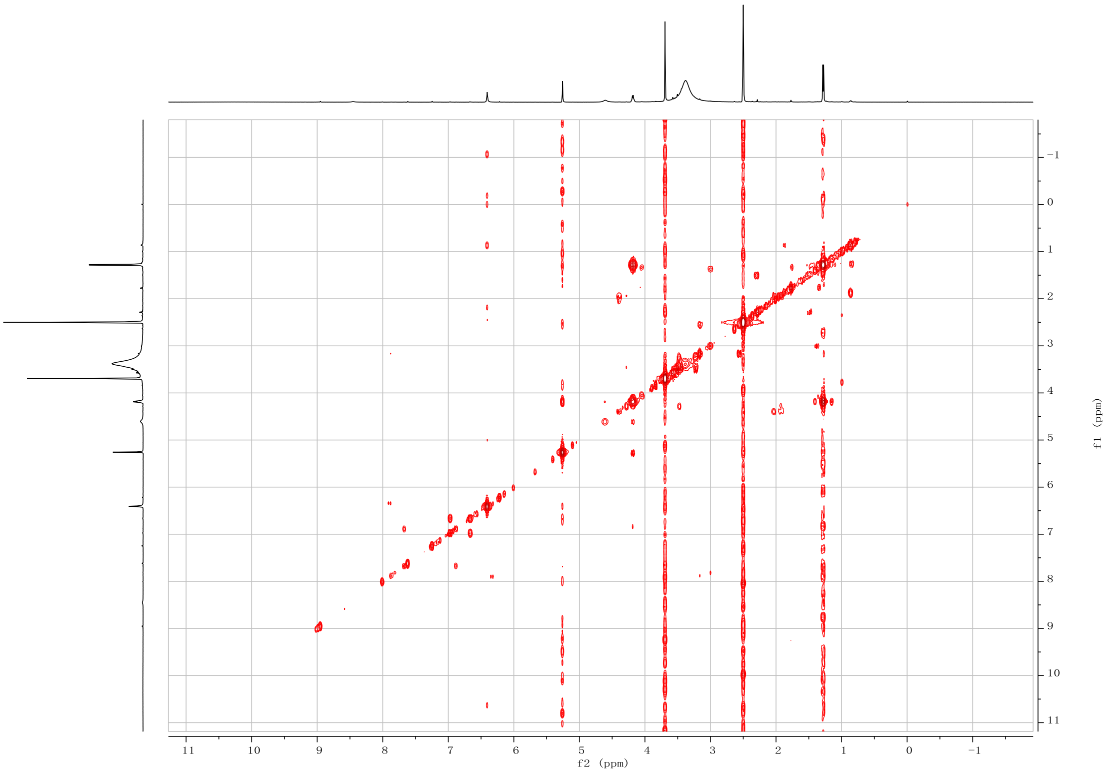

Figure S5: COSY spectrum of **1** in DMSO-d_6.

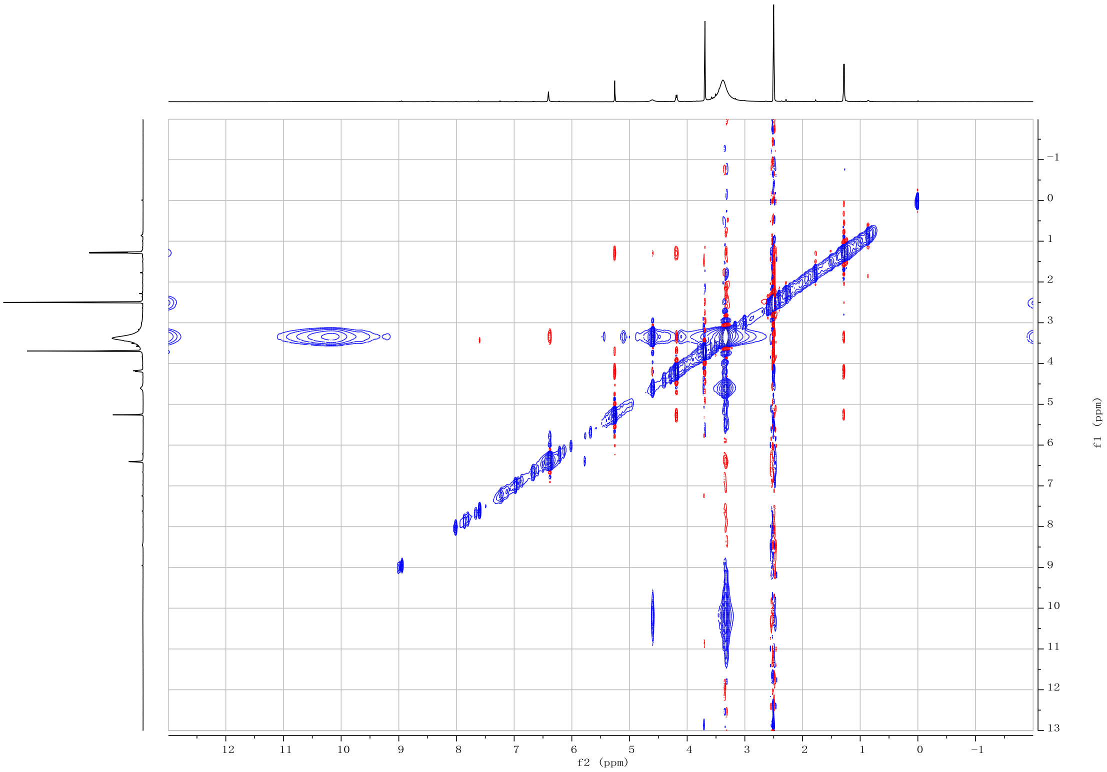

Figure S6: NOESY spectrum of **1** in DMSO-d_6.

Generic Display Report

Analysis Info

Analysis Name	D:\Data\MS\data\202310\caijian_2MB1_pos_81_01_16018.d	Acquisition Date	10/7/2023 3:22:20 PM
Method	LC_Direct Infusion_pos_70-500mz.m	Operator	SCSIO
Sample Name	caijian_2MB1_pos	Instrument	maXis
Comment			

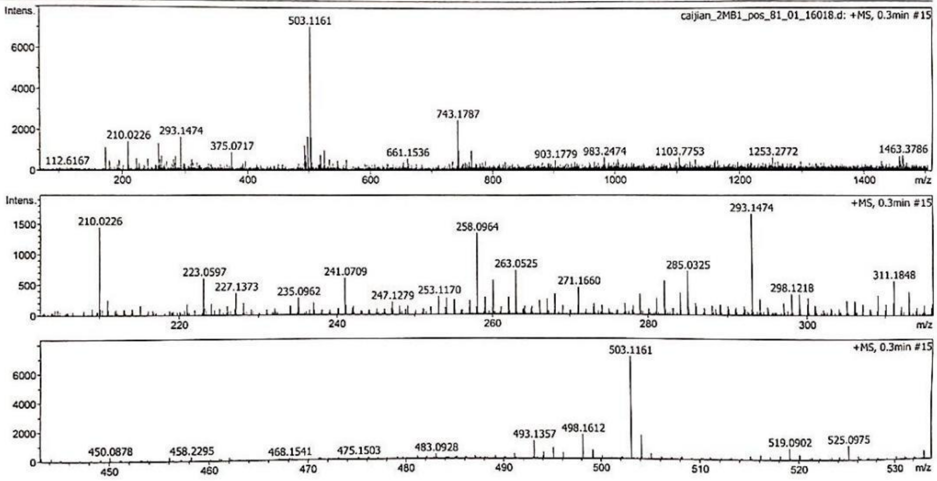

Bruker Compass DataAnalysis 4.1 printed: 10/7/2023 3:27:57 PM by: SCSIO Page 1 of 1

Mass Spectrum SmartFormula Report

Analysis Info

Analysis Name	D:\Data\MS\data\202310\caijian_2MB1_pos_81_01_16018.d	Acquisition Date	10/7/2023 3:22:20 PM	
Method	LC_Direct Infusion_pos_70-500mz.m	Operator	SCSIO	
Sample Name	caijian_2MB1_pos	Instrument	maXis	255552.00029
Comment				

Acquisition Parameter

Source Type	ESI	Ion Polarity	Positive	Set Nebulizer	0.4 Bar
Focus	Active	Set Capillary	4500 V	Set Dry Heater	180 °C
Scan Begin	70 m/z	Set End Plate Offset	-500 V	Set Dry Gas	4.0 l/min
Scan End	1500 m/z	Set Charging Voltage	0 V	Set Divert Valve	Waste
		Set Corona	0 nA	Set APCI Heater	0 °C

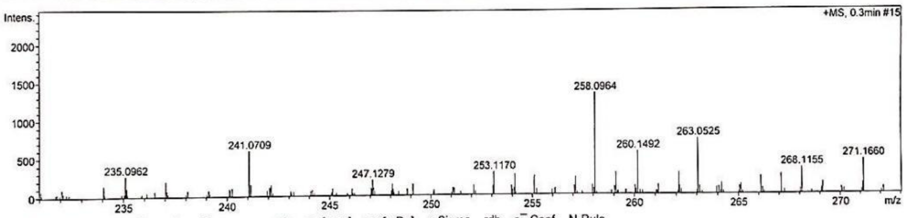

Meas. m/z	#	Ion Formula	Score	m/z	err [ppm]	err [mDa]	mSigma	rdb	e⁻ Conf	N-Rule
241.0709	1	C11H13O6	100.00	241.0707	0.8	0.2	38.5	5.5	even	ok
263.0525	1	C11H12NaO6	100.00	263.0526	0.3	0.1	12.1	5.5	even	ok

caijian_2MB1_pos_81_01_16018.d
Bruker Compass DataAnalysis 4.1 printed: 10/7/2023 3:27:25 PM by: SCSIO Page 1 of 1

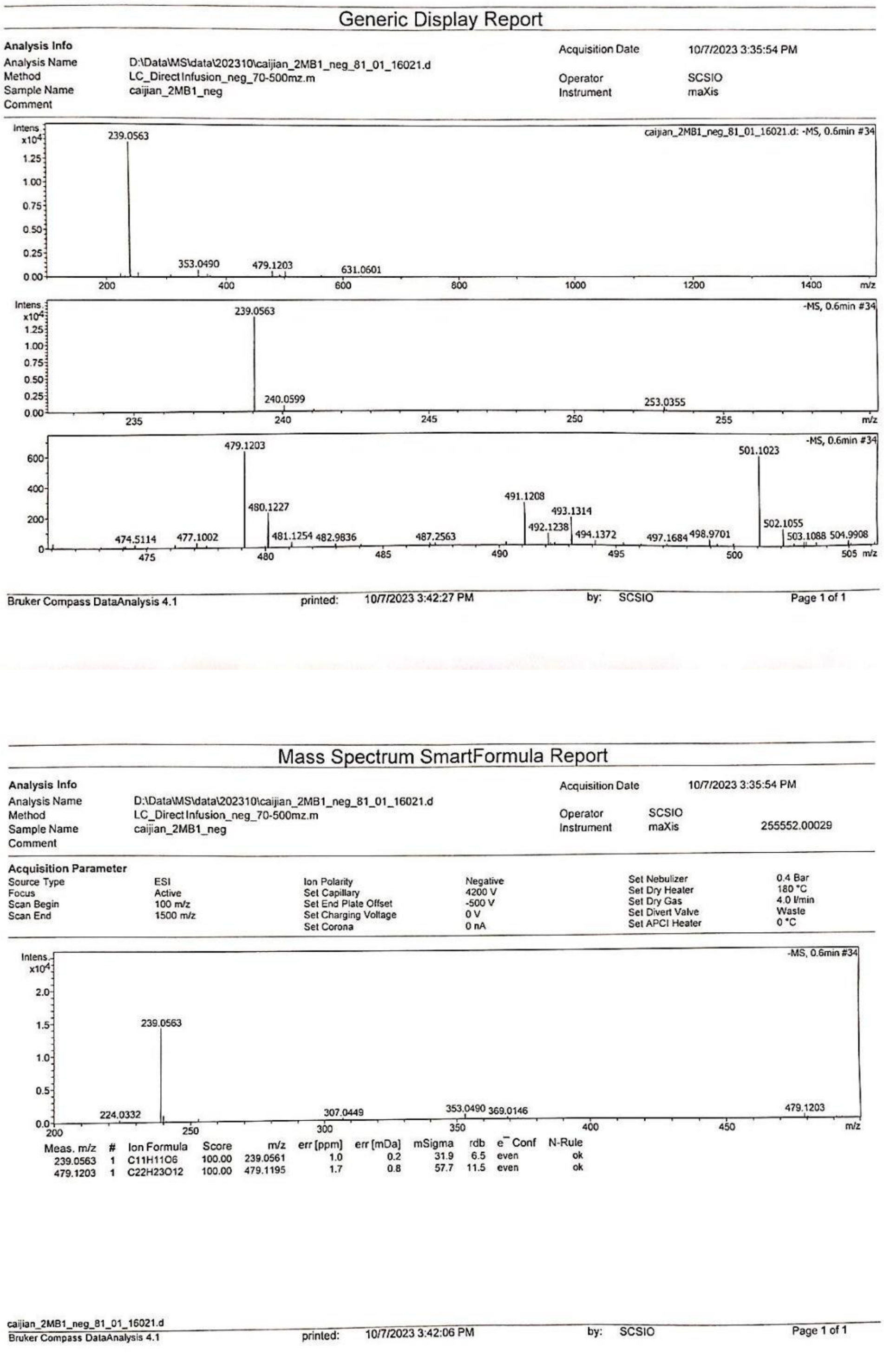

Figure S7: HRESIMS spectrum of **1**.

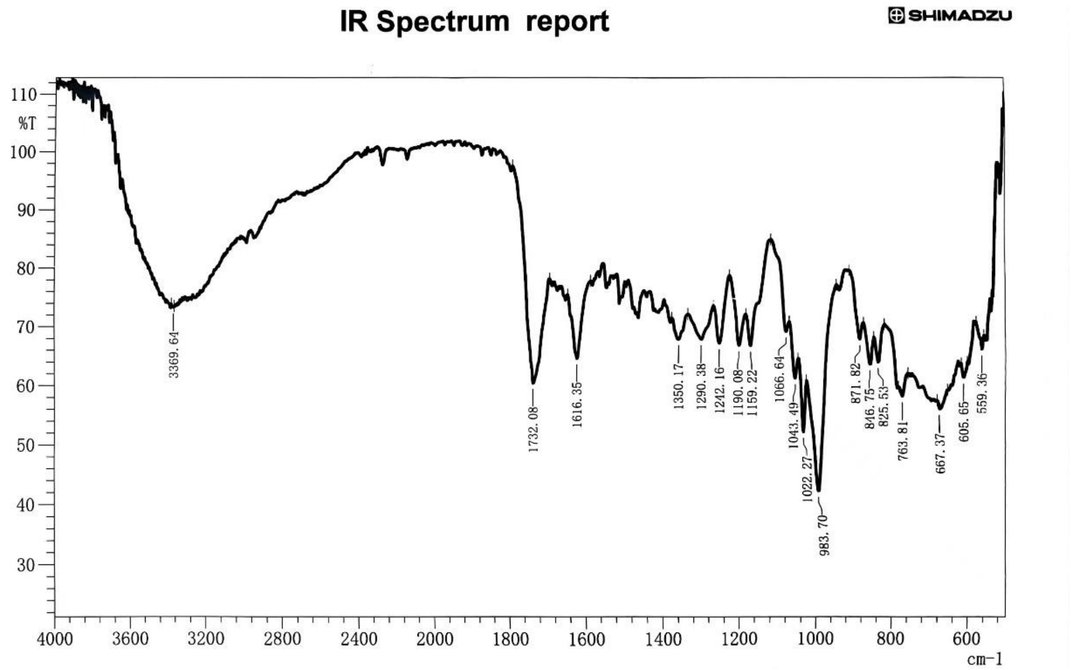

Figure S8: IR spectrum of **1**.

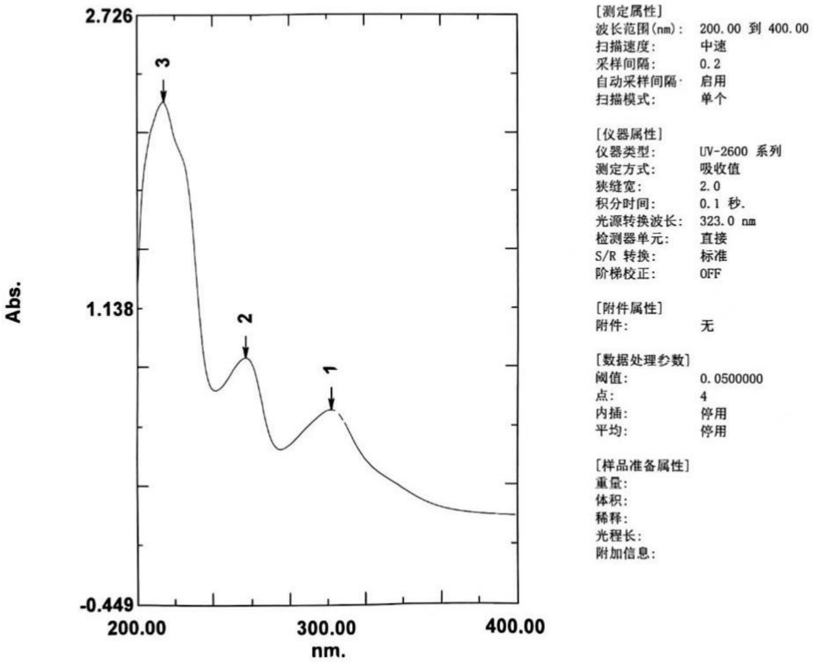

No.	P/V	波长(nm)	Abs.	描述
1	●	301.80	0.591	
2	●	257.20	0.868	
3	●	213.80	2.257	

Figure S9: UV spectrum of **1** in MeOH.

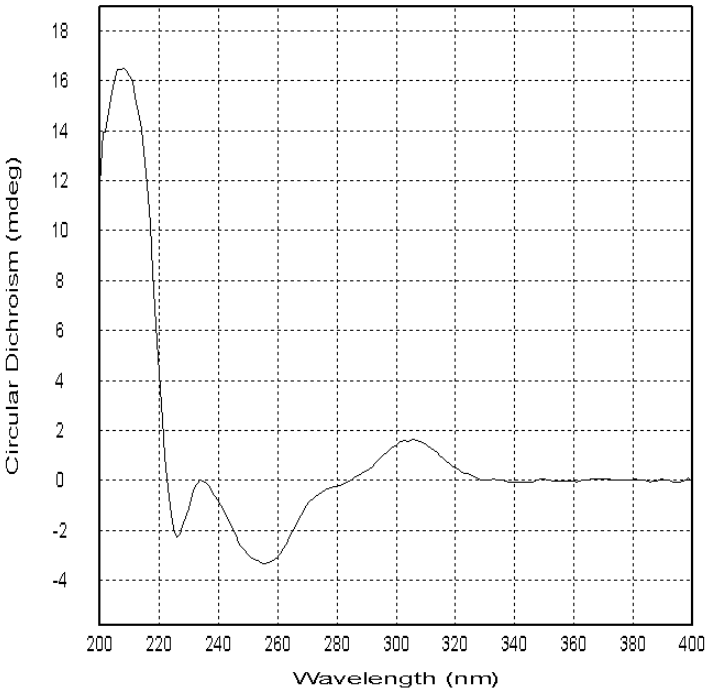

Figure S10: ECD spectrum of **1** in MeOH.

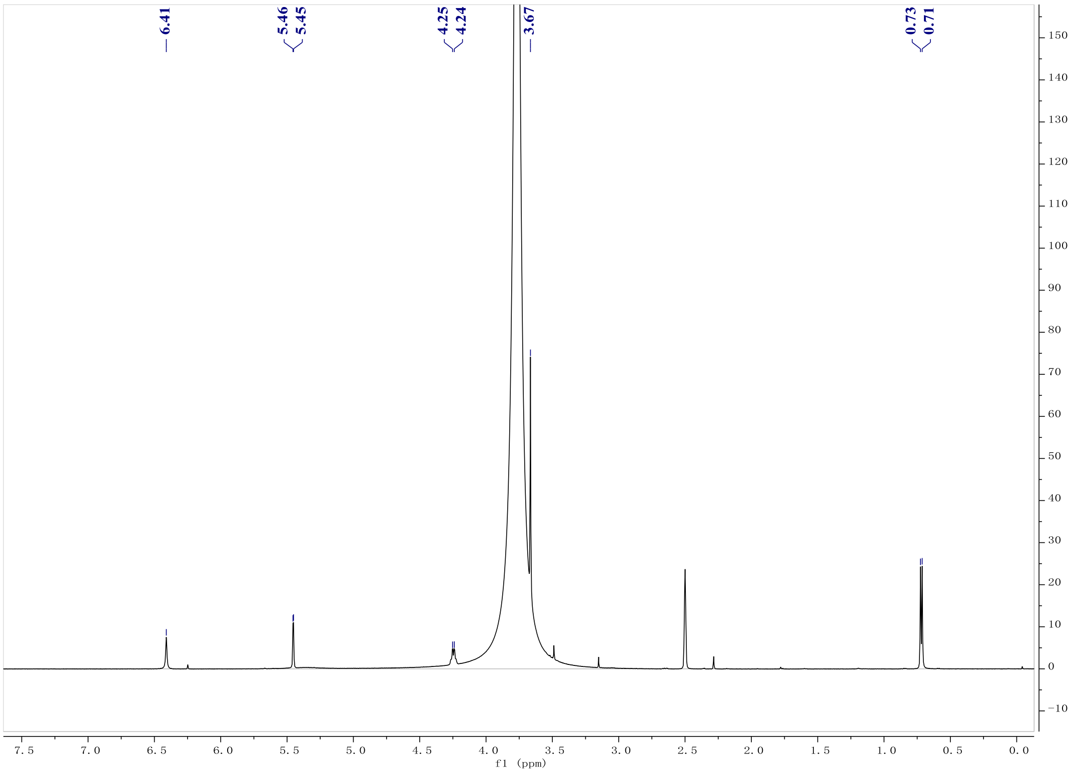

Figure S11: ^{1}H NMR spectrum of **2** in DMSO-d_6.

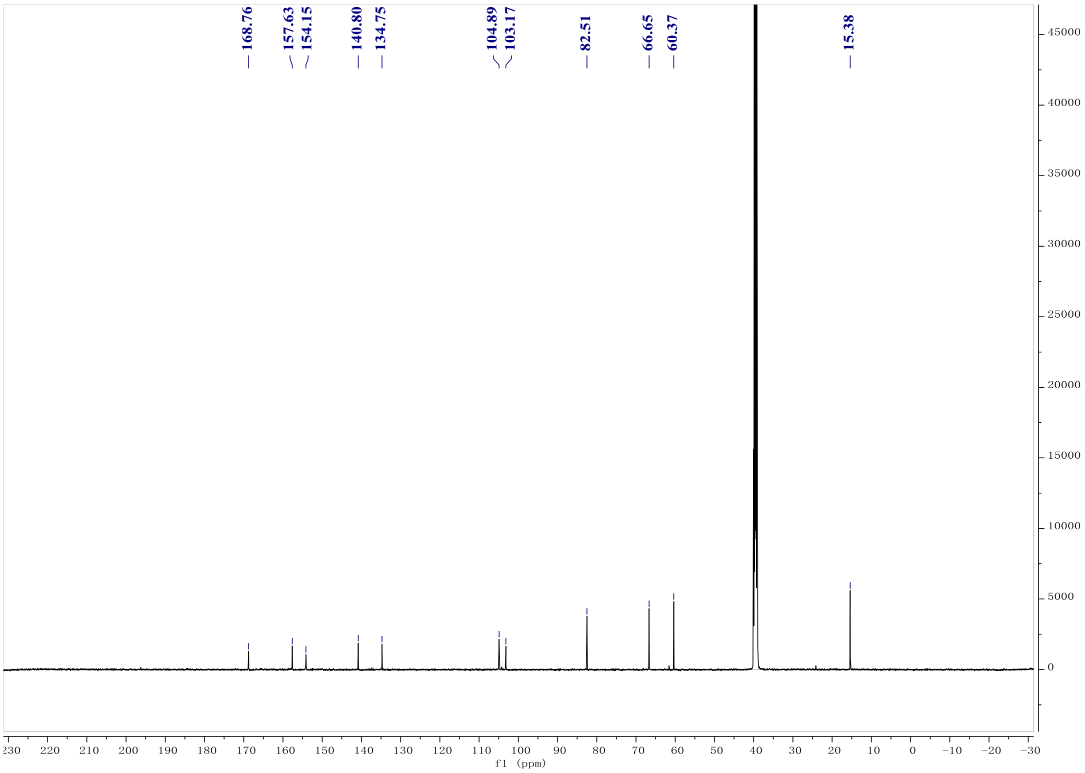

Figure S12: ^{13}C NMR spectrum of **2** in DMSO-d_6.

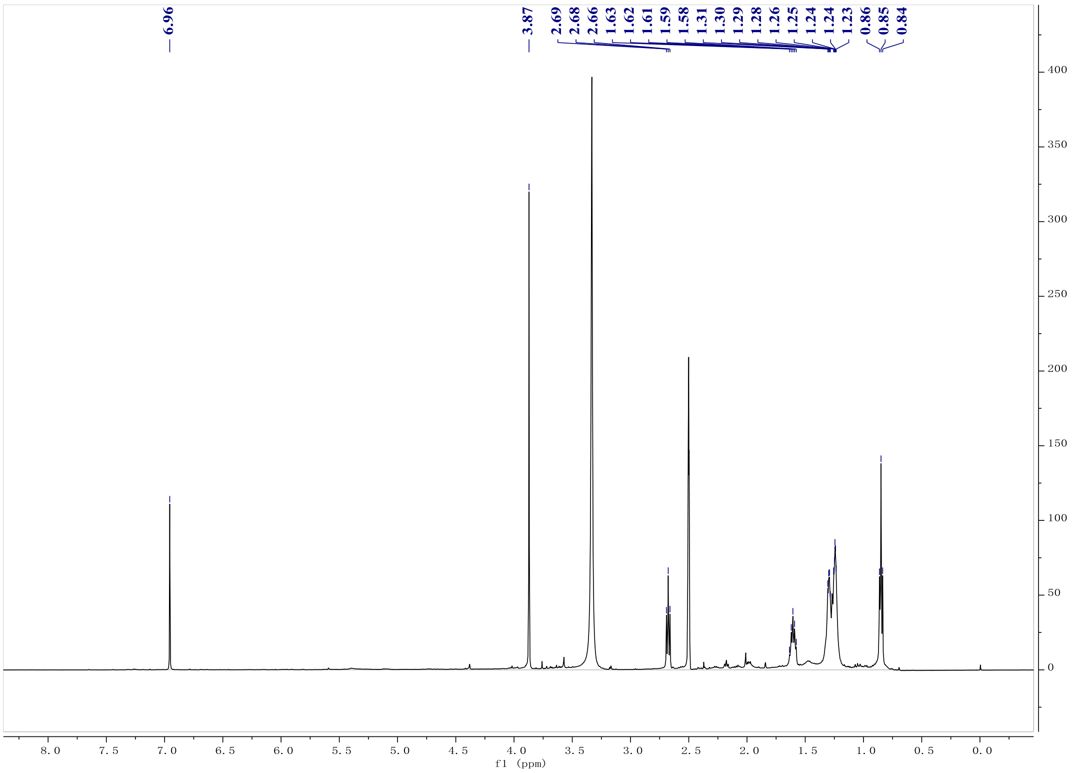

Figure S13: ^{1}H NMR spectrum of **3** in DMSO-d_6.

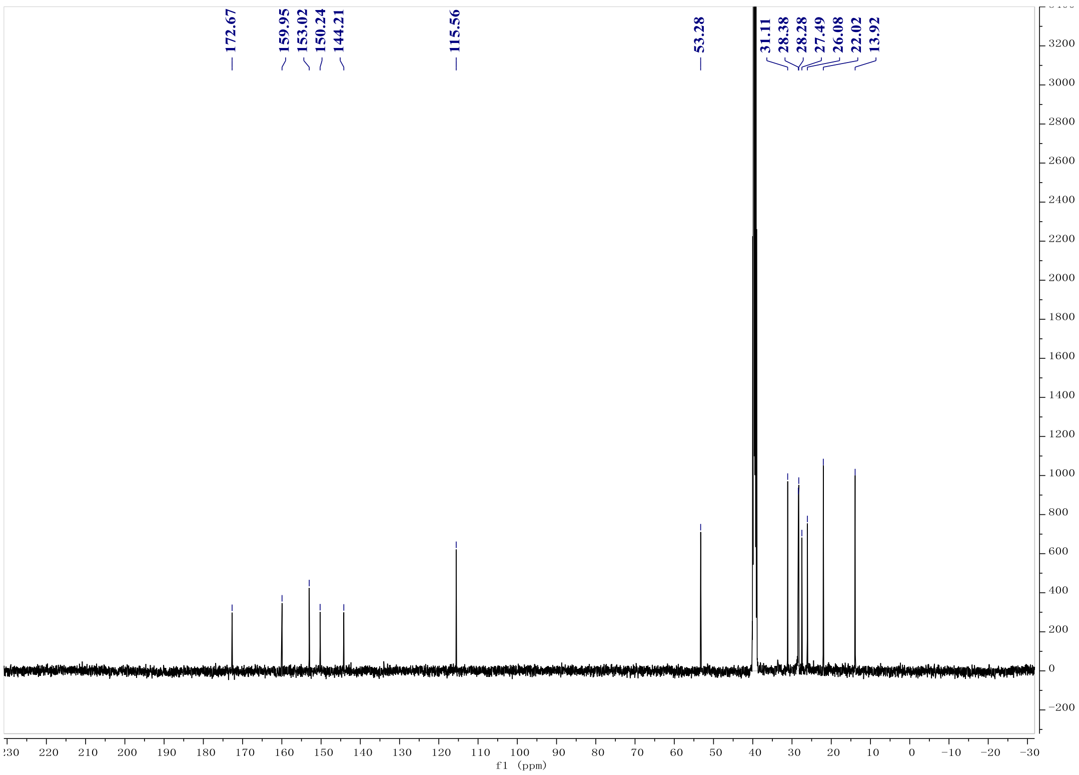

Figure S14: ^{13}C NMR spectrum of **3** in DMSO-d_6.

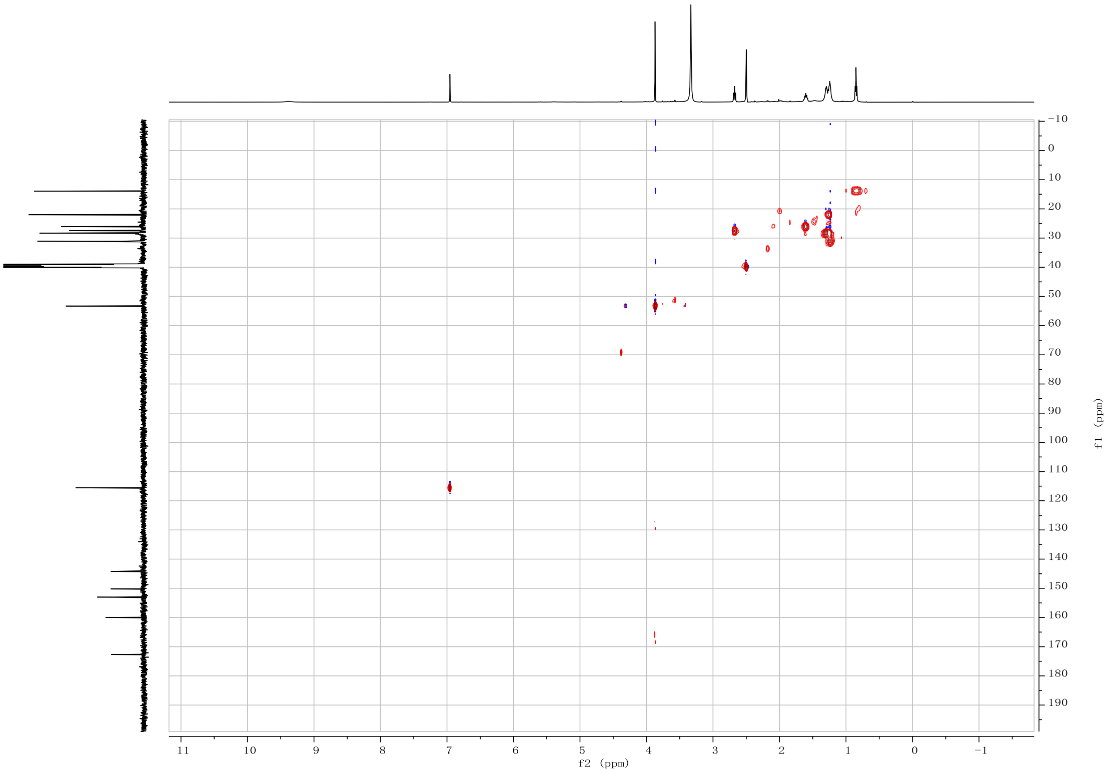

Figure S15: HSQC spectrum of **3** in DMSO-d_6.

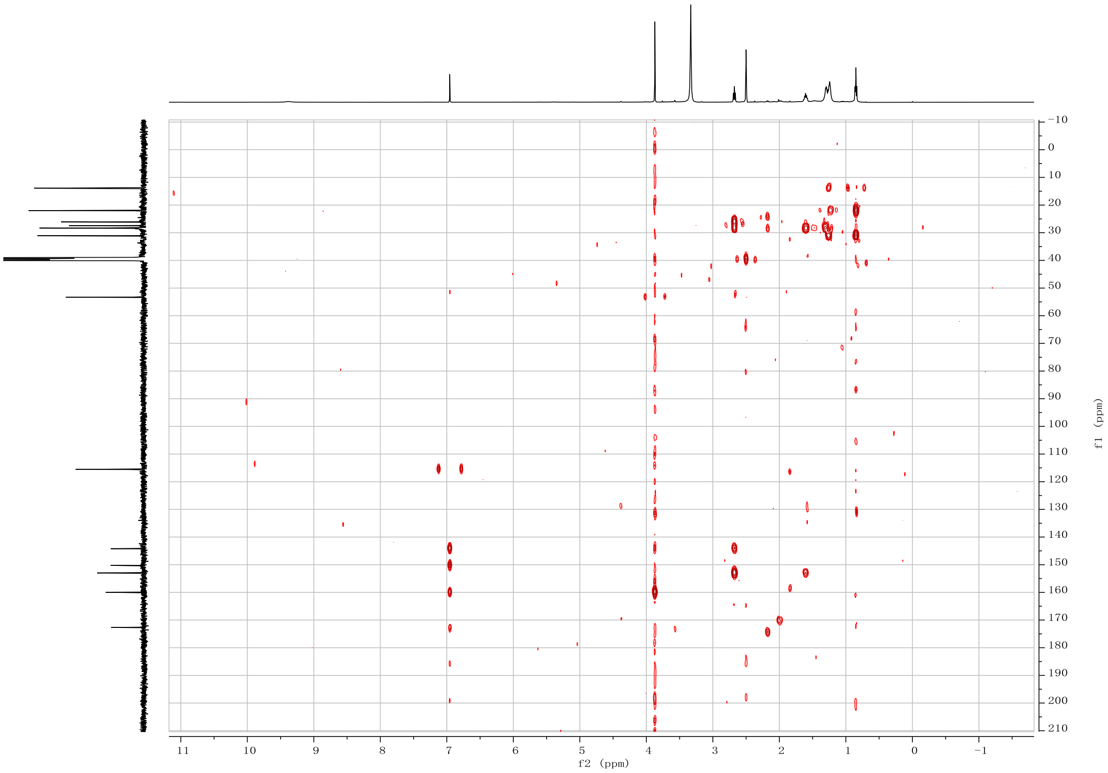

Figure S16: HMBC spectrum of **3** in DMSO-d_6.

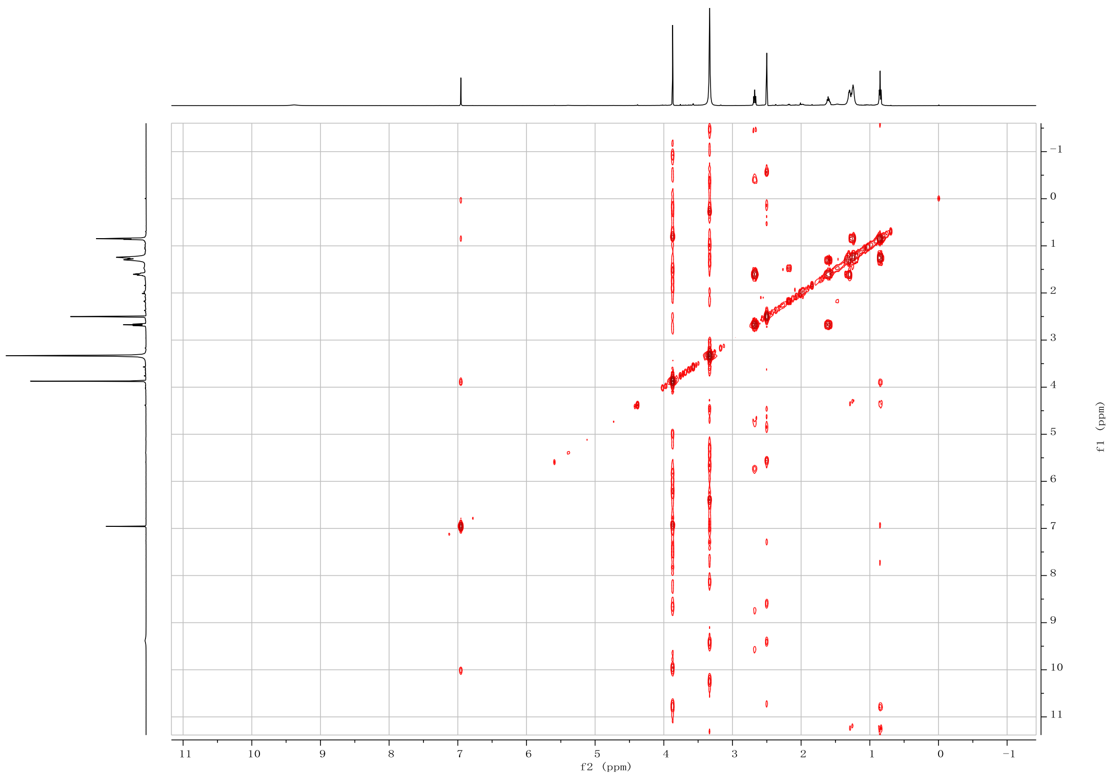

Figure S17: COSY spectrum of **3** in DMSO-*d*6.

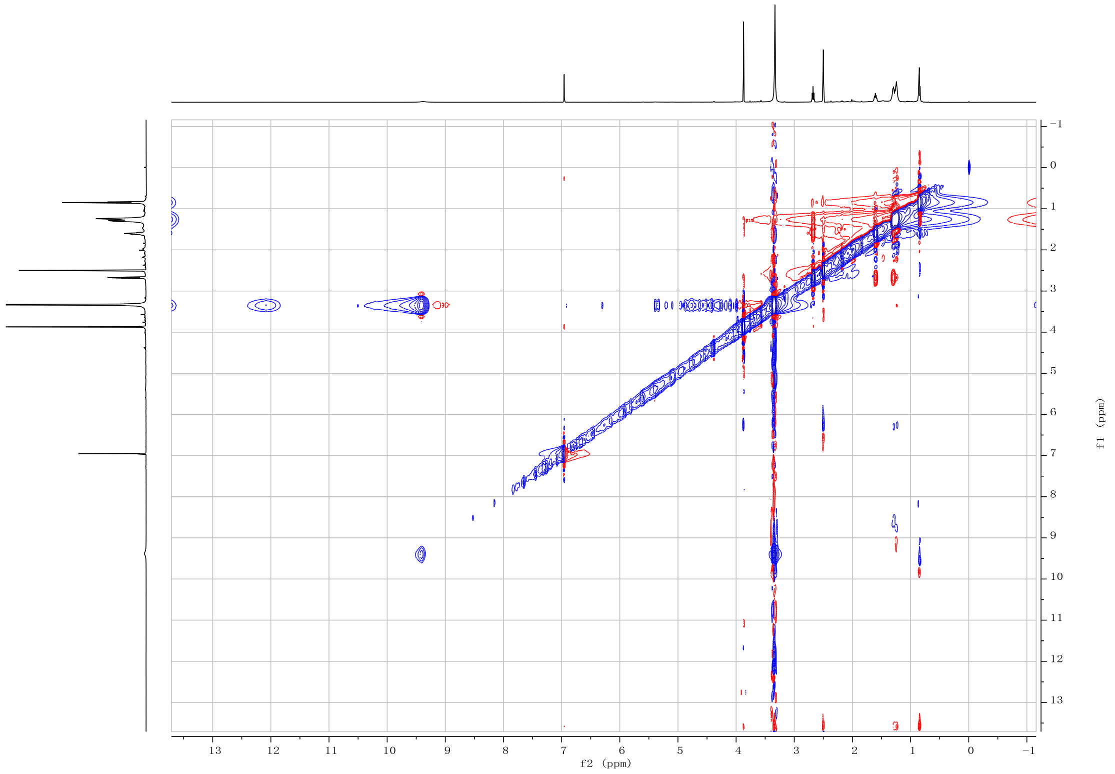

Figure S18: NOESY spectrum of **3** in DMSO-*d*6.

Mass Spectrum SmartFormula Report

Analysis Info

Analysis Name	D:\Data\MS\data\202312\chenyi_2MB12_pos_82_01_16500.d	Acquisition Date	12/26/2023 12:19:17 PM
Method	LC_Direct Infusion_pos_70-500mz.m	Operator	SCSIO
Sample Name	chenyi_2MB12_pos	Instrument	maXis 255552.00029
Comment			

Acquisition Parameter

Source Type	ESI	Ion Polarity	Positive	Set Nebulizer	0.4 Bar
Focus	Active	Set Capillary	4500 V	Set Dry Heater	180 °C
Scan Begin	70 m/z	Set End Plate Offset	-500 V	Set Dry Gas	4.0 l/min
Scan End	1500 m/z	Set Charging Voltage	0 V	Set Divert Valve	Waste
		Set Corona	0 nA	Set APCI Heater	0 °C

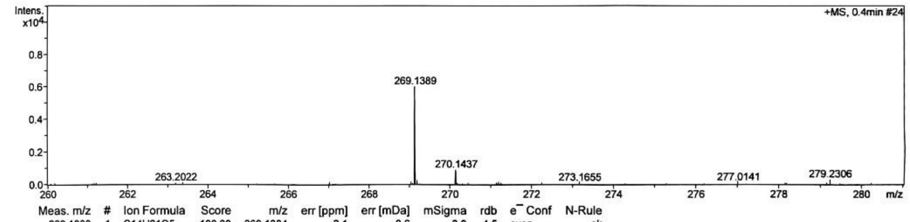

Meas. m/z	#	Ion Formula	Score	m/z	err [ppm]	err [mDa]	mSigma	rdb	e⁻ Conf	N-Rule
269.1389	1	C14H21O5	100.00	269.1384	2.1	0.6	3.9	4.5	even	ok

chenyi_2MB12_pos_82_01_16500.d

Bruker Compass DataAnalysis 4.1 printed: 12/26/2023 5:19:46 PM by: SCSIO Page 1 of 1

Figure S19: HRESIMS spectrum of **3**.

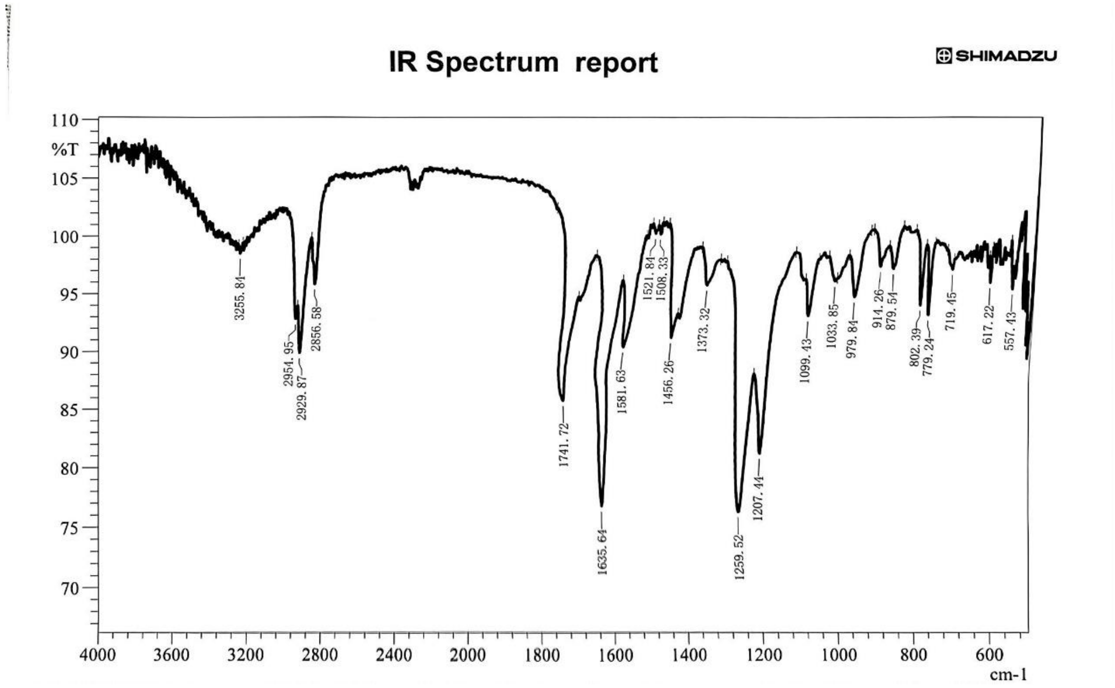

Figure S20: IR spectrum of **3**.

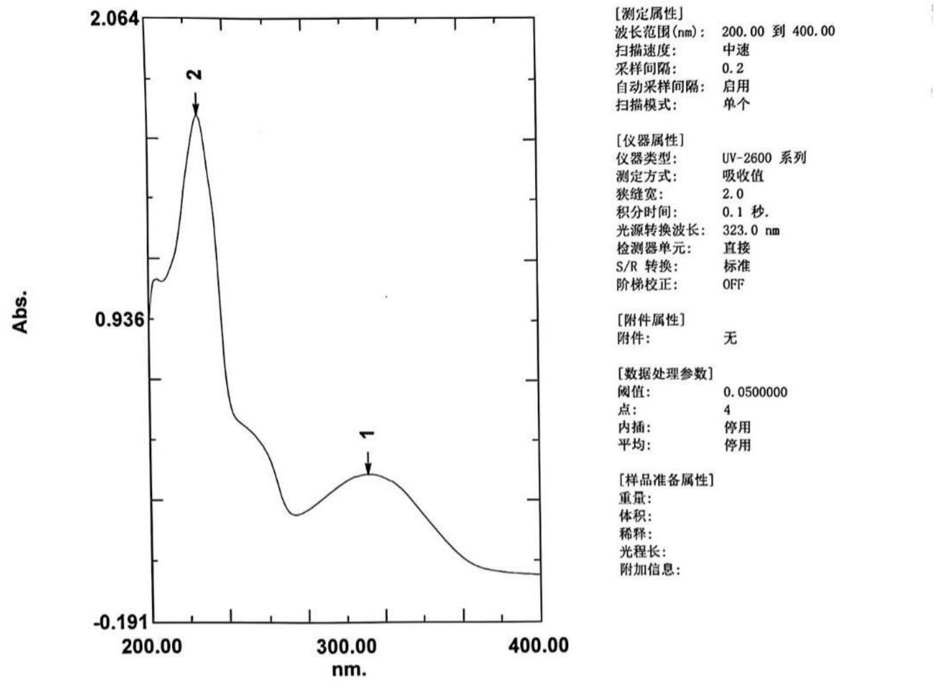

No.	P/V	波长(nm)	Abs.	描述
1	⊕	311.60	0.356	
2	⊕	225.60	1.703	

Figure S21: UV spectrum of **3** in MeOH.

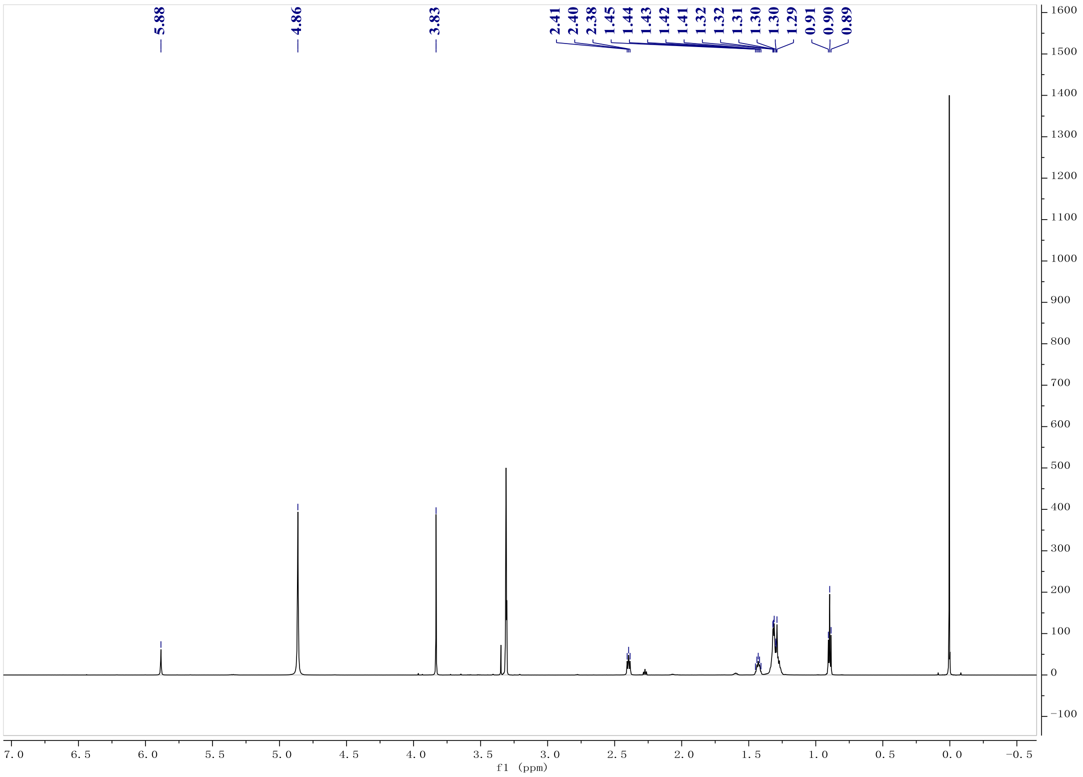

Figure S22: ^{1}H NMR spectrum of **4** in Methanol-d_4.

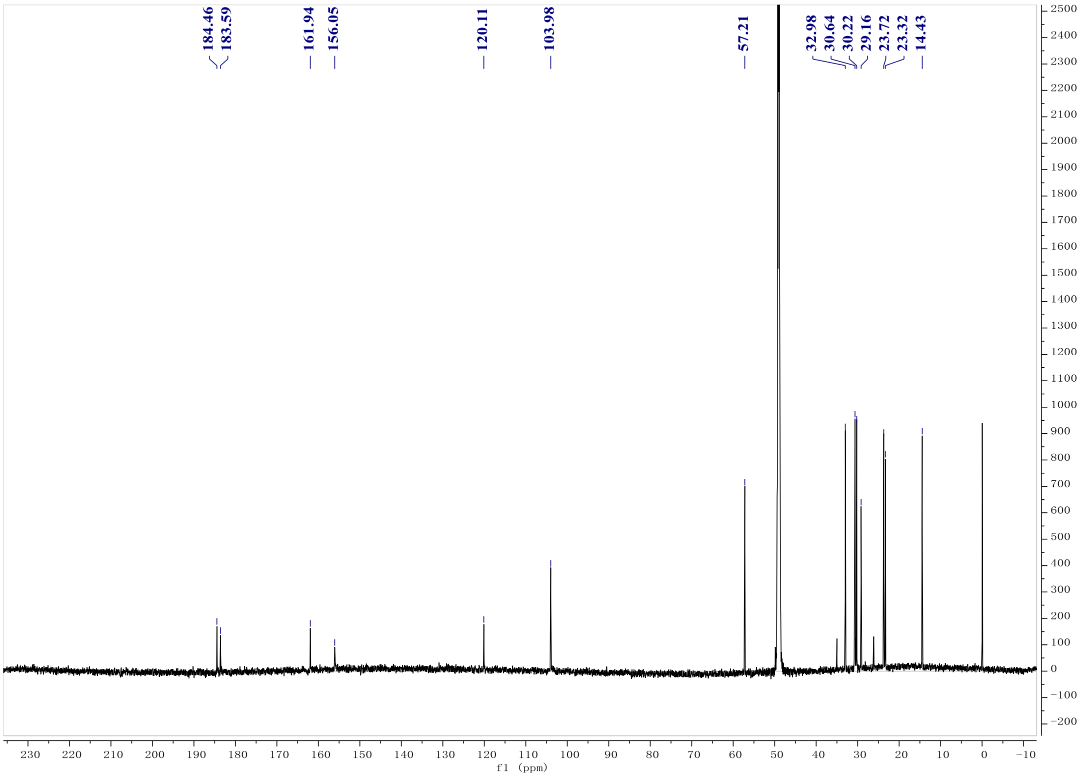

Figure S23: ^{13}C NMR spectrum of **4** in Methanol-d_4.

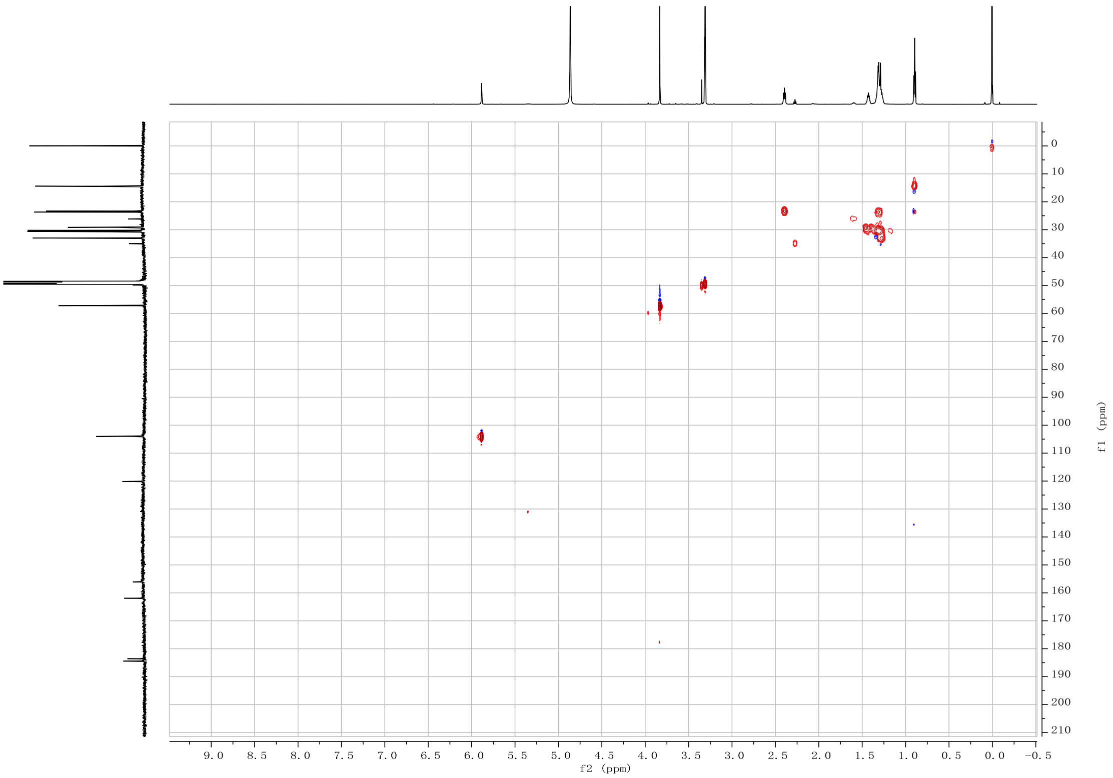

Figure S24: HSQC spectrum of **4** in Methanol-d_4.

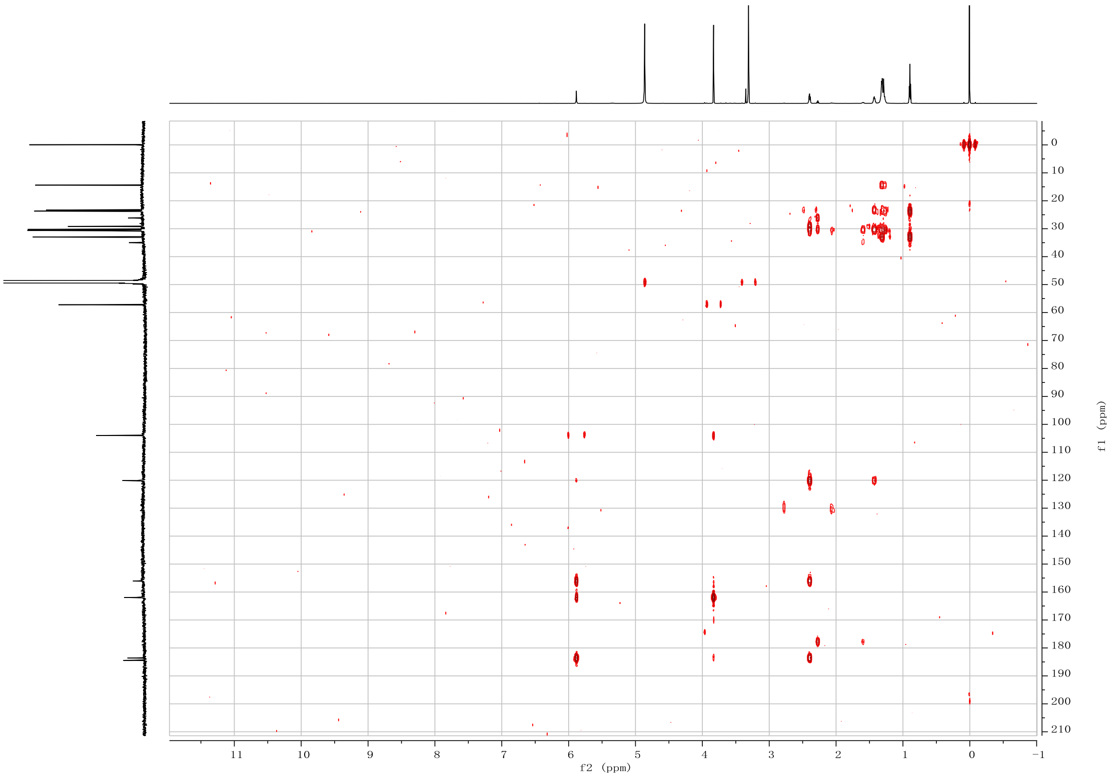

Figure S25: HMBC spectrum of **4** in Methanol-d_4.

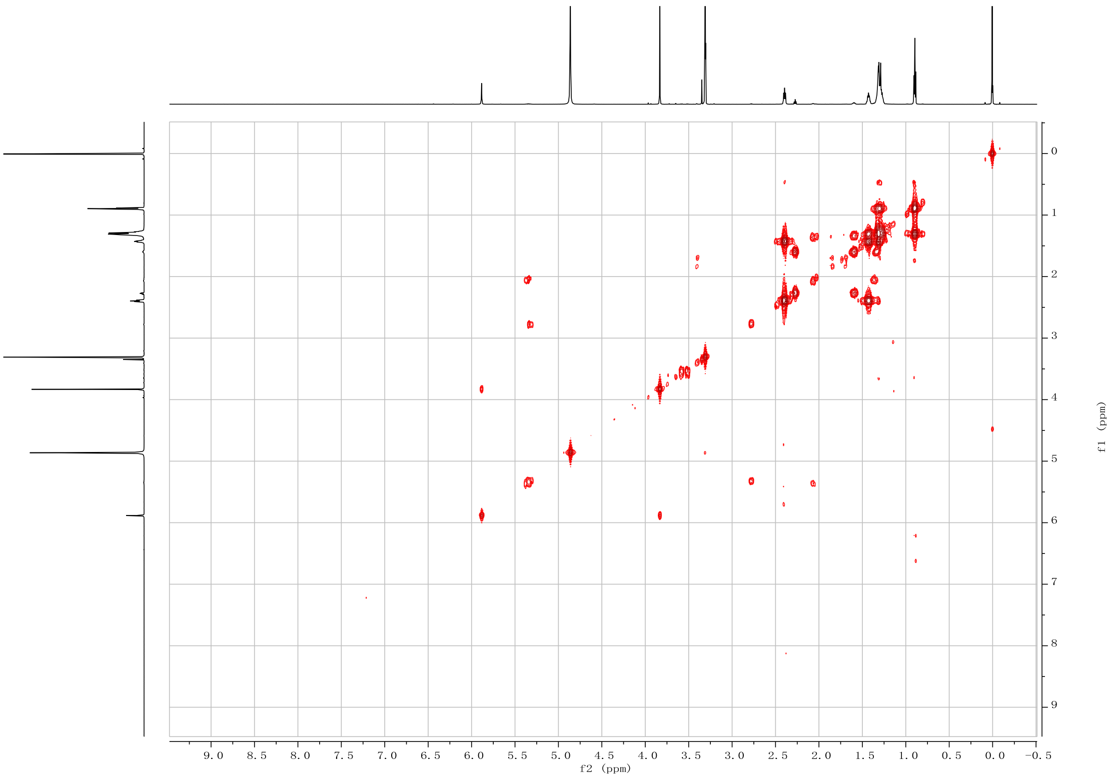

Figure S26: COSY spectrum of **4** in Methanol-d_4.

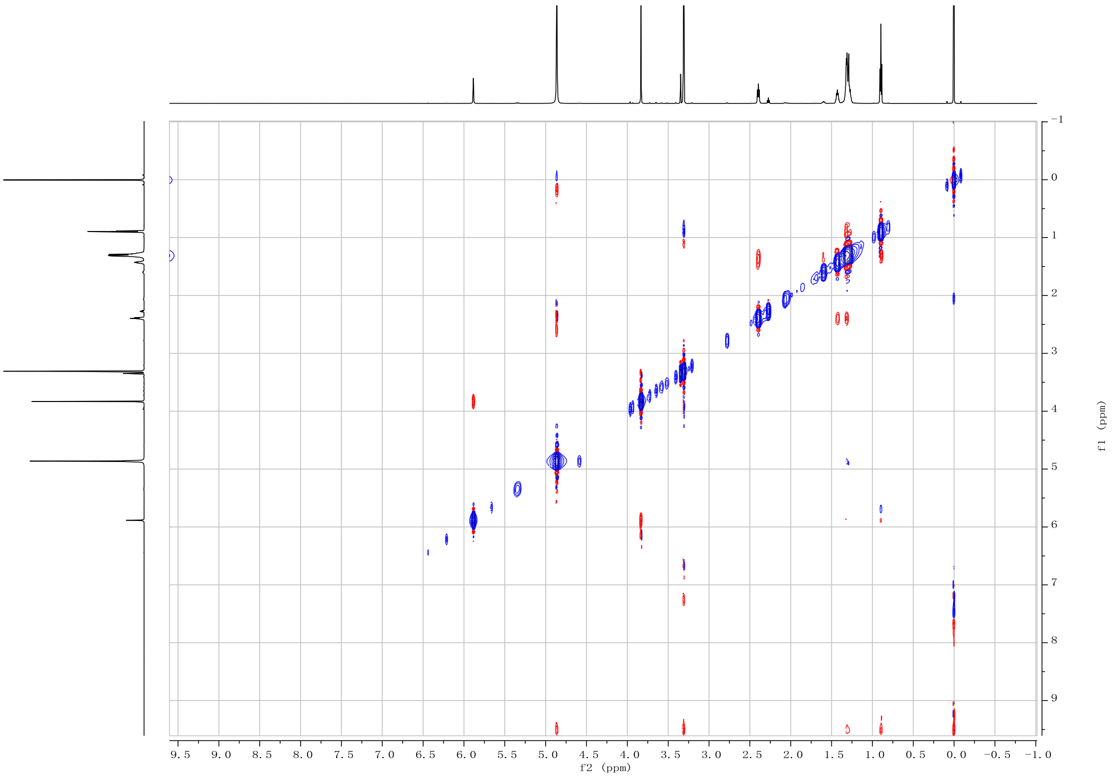

Figure S27: NOESY spectrum of **4** in Methanol-d_4.

Generic Display Report

Analysis Info

Analysis Name	D:\Data\MS\data\202401\chenyi_2MB17-a_pos_82_01_16638.d	Acquisition Date	1/16/2024 11:59:35 AM
Method	LC_Direct Infusion_pos_70-500mz.m	Operator	SCSIO
Sample Name	chenyi_2MB17-a_pos	Instrument	maXis
Comment			

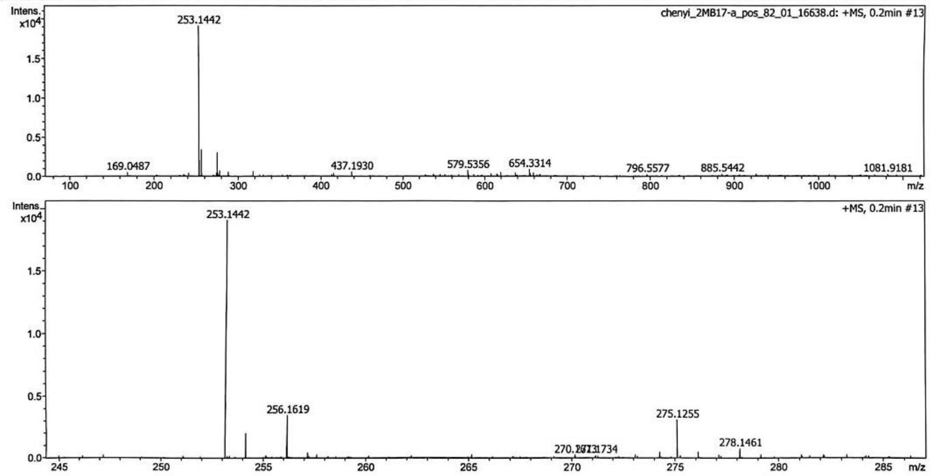

Bruker Compass DataAnalysis 4.1 printed: 1/16/2024 12:06:08 PM by: SCSIO Page 1 of 1

Mass Spectrum SmartFormula Report

Analysis Info

Analysis Name	D:\Data\MS\data\202401\chenyi_2MB17-a_pos_82_01_16638.d	Acquisition Date	1/16/2024 11:59:35 AM	
Method	LC_Direct Infusion_pos_70-500mz.m	Operator	SCSIO	
Sample Name	chenyi_2MB17-a_pos	Instrument	maXis	255552.00029
Comment				

Acquisition Parameter

Source Type	ESI	Ion Polarity	Positive	Set Nebulizer	0.4 Bar
Focus	Active	Set Capillary	4500 V	Set Dry Heater	180 °C
Scan Begin	70 m/z	Set End Plate Offset	-500 V	Set Dry Gas	4.0 l/min
Scan End	1500 m/z	Set Charging Voltage	0 V	Set Divert Valve	Waste
		Set Corona	0 nA	Set APCI Heater	0 °C

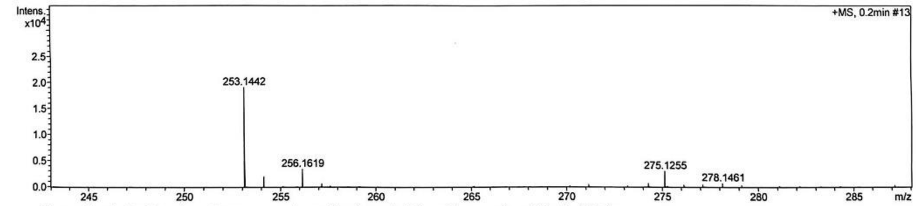

Meas. m/z	#	Ion Formula	Score	m/z	err [ppm]	err [mDa]	mSigma	rdb	e⁻ Conf	N-Rule
253.1442	1	C14H21O4	100.00	253.1434	-3.1	-0.8	28.6	4.5	even	ok
275.1255	1	C14H20NaO4	100.00	275.1254	0.6	0.2	12.0	4.5	even	ok

chenyi_2MB17-a_pos_82_01_16638.d
Bruker Compass DataAnalysis 4.1 printed: 1/16/2024 12:05:46 PM by: SCSIO Page 1 of 1

Figure S28: HRESIMS spectrum of **4**.

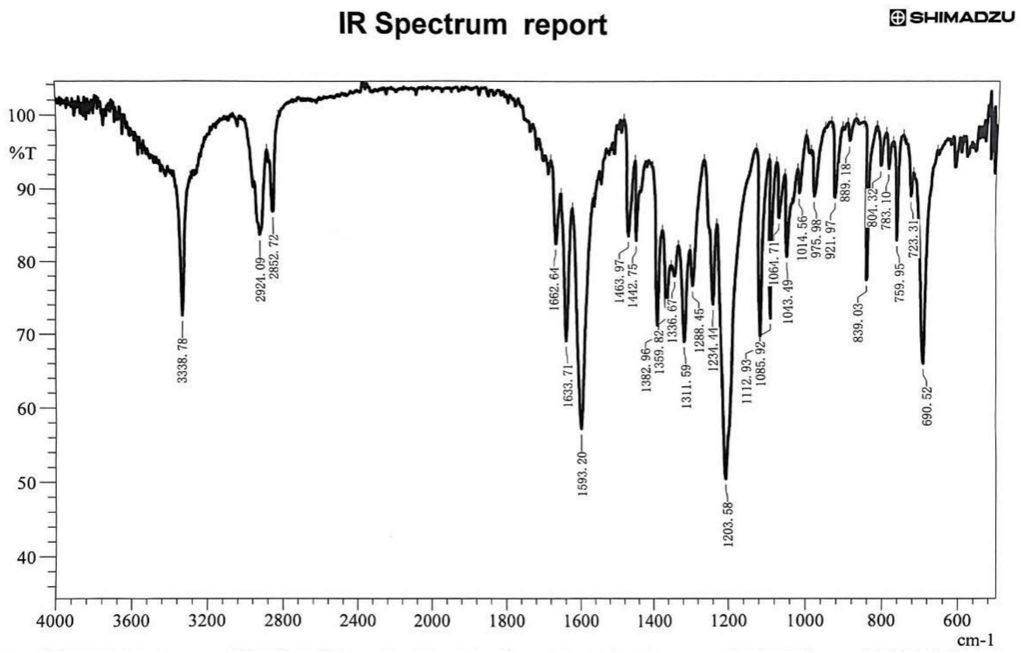

Figure S29: IR spectrum of **4**.

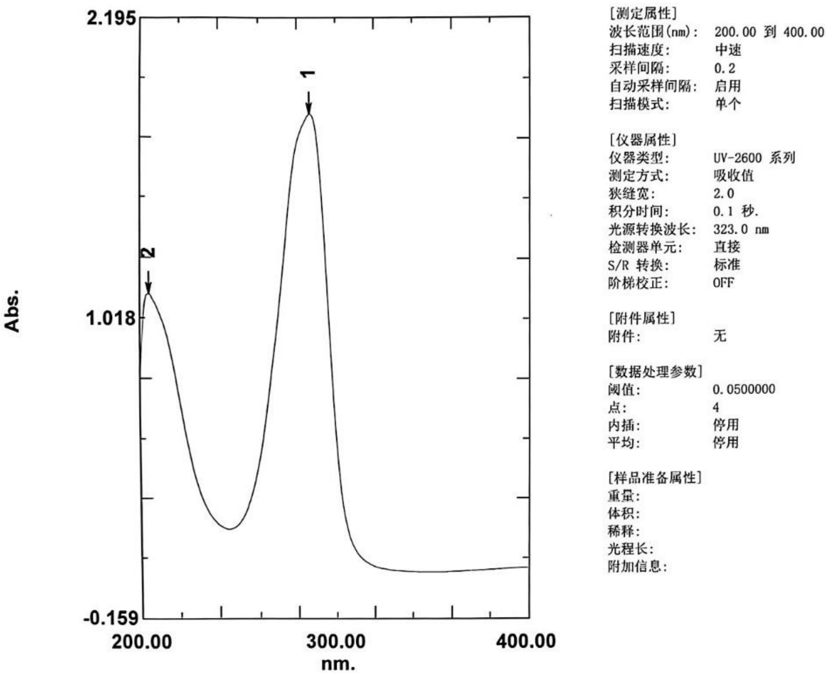

No.	P/V	波长(nm)	Abs.	描述
1	⬆	286.80	1.818	
2	⬆	204.60	1.114	

Figure S30: UV spectrum of **4** in MeOH.

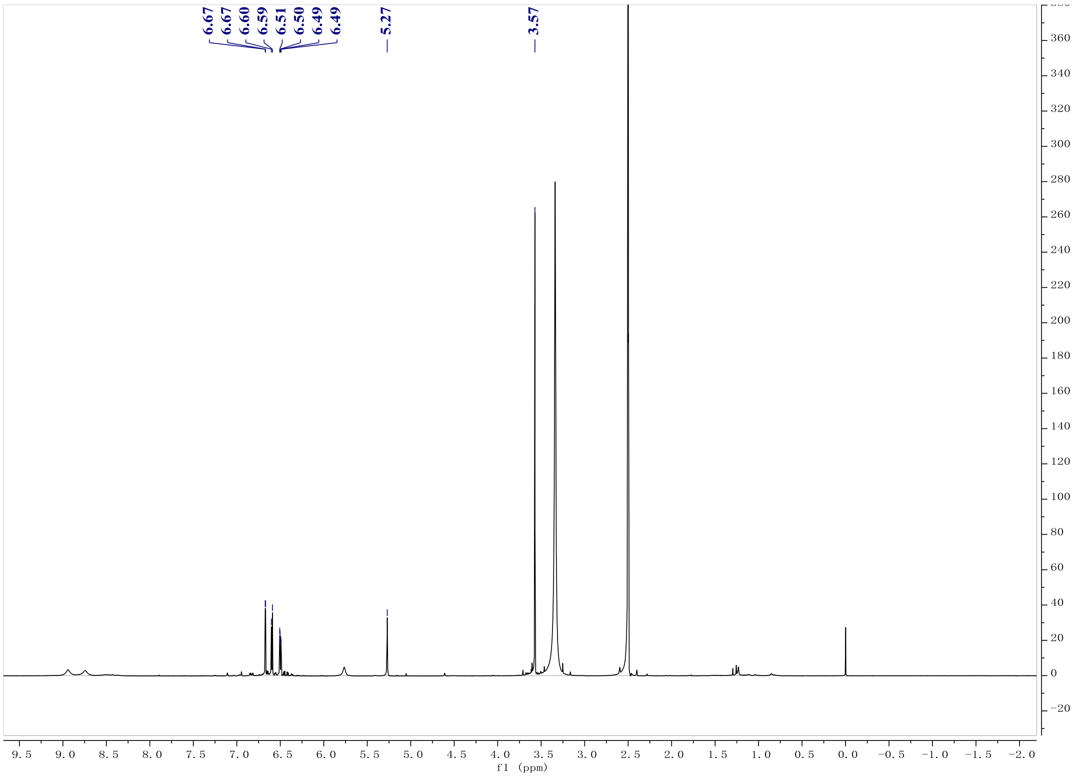

Figure S31: ^{1}H NMR spectrum of **5** in DMSO-d_6.

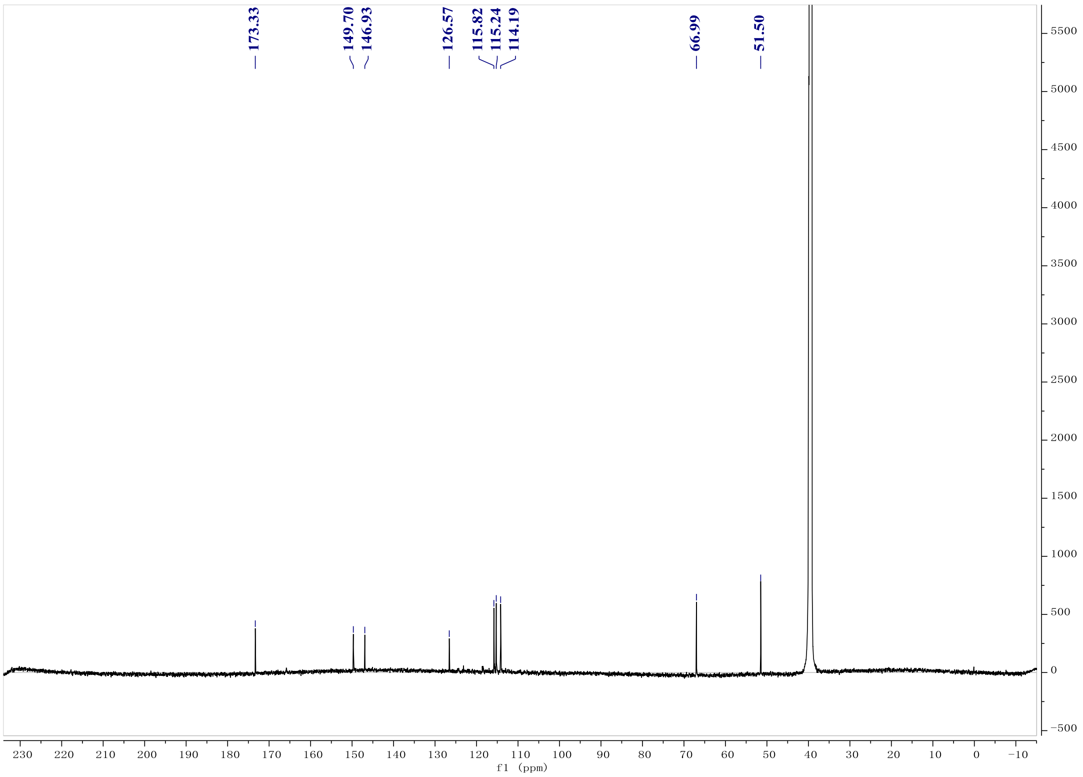

Figure S32: ^{13}C NMR spectrum of **5** in DMSO-d_6.

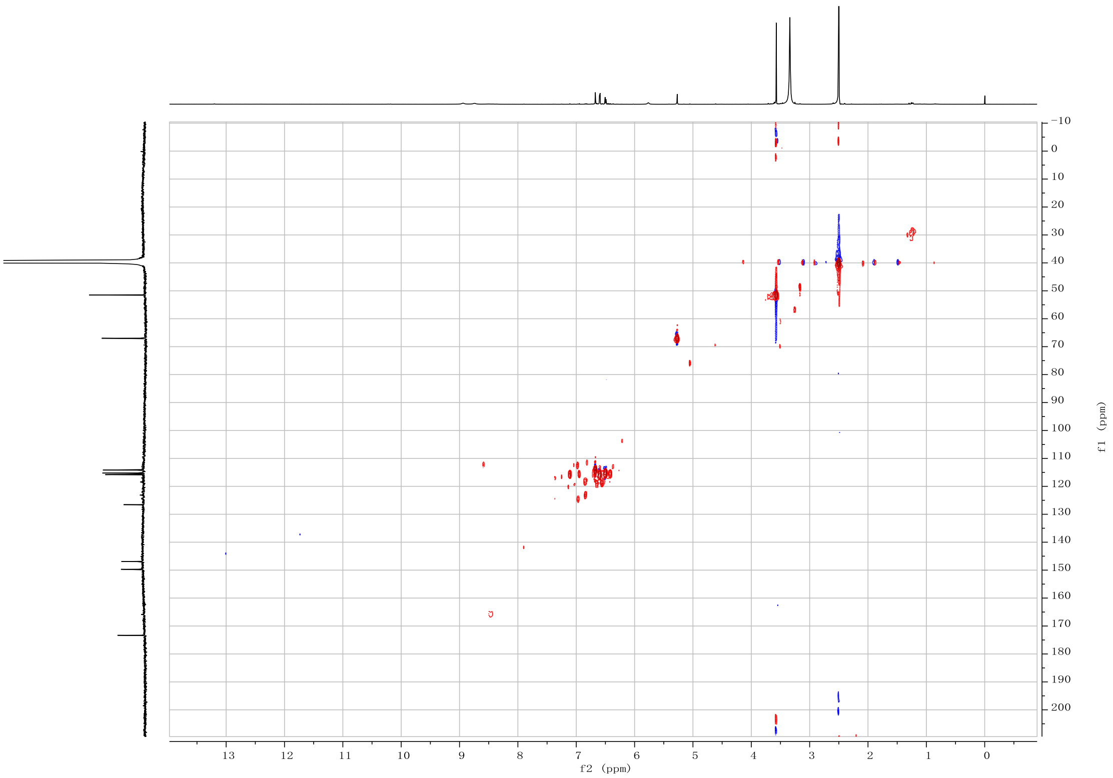

Figure S33: HSQC spectrum of **5** in DMSO-d_6.

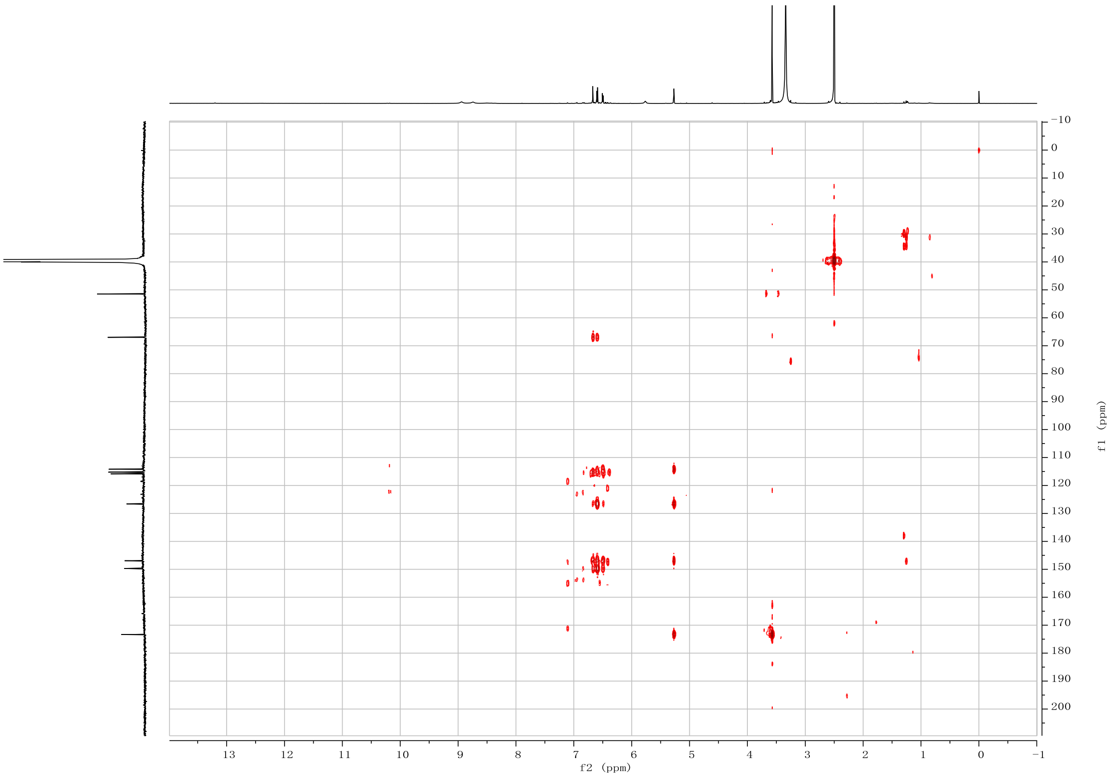

Figure S34: HMBC spectrum of **5** in DMSO-d_6.

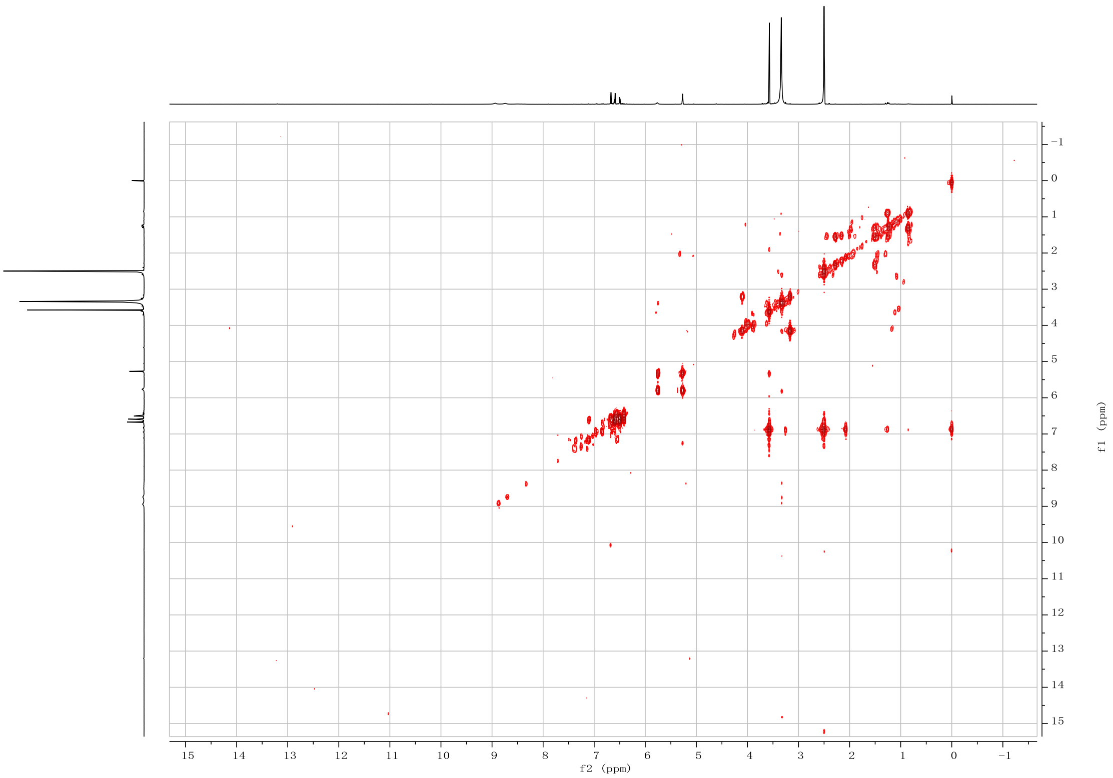

Figure S35: COSY spectrum of **5** in DMSO-d_6.

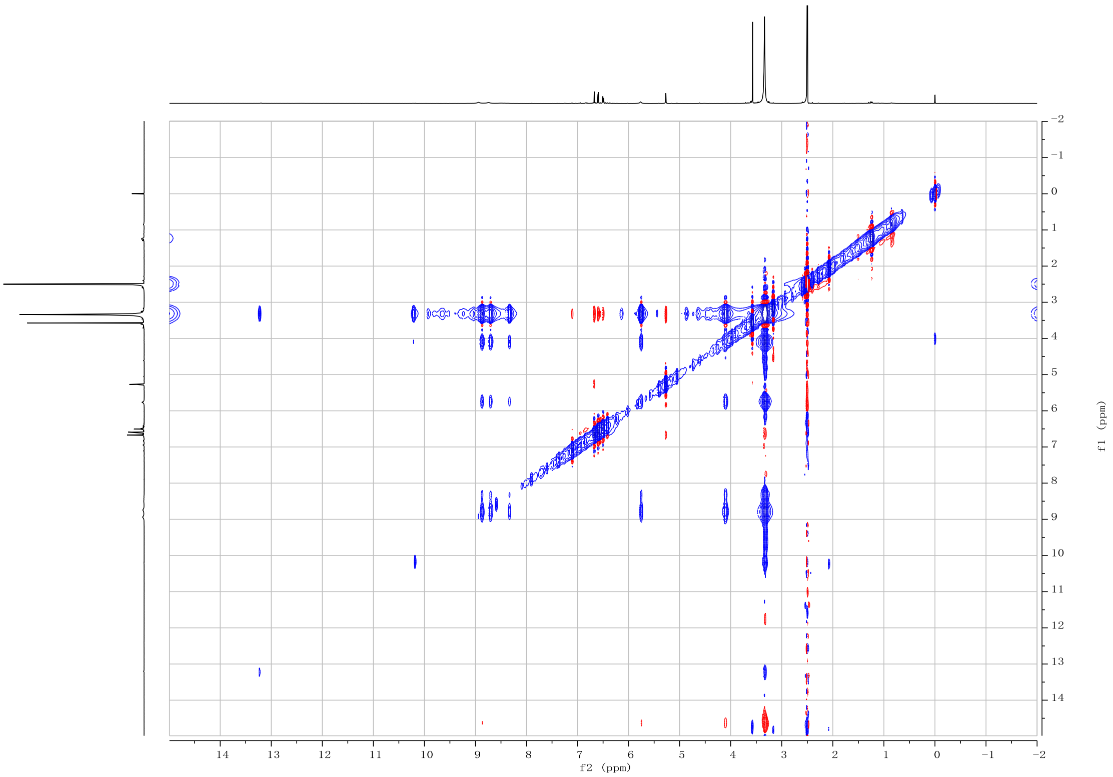

Figure S36: NOESY spectrum of **5** in DMSO-d_6.

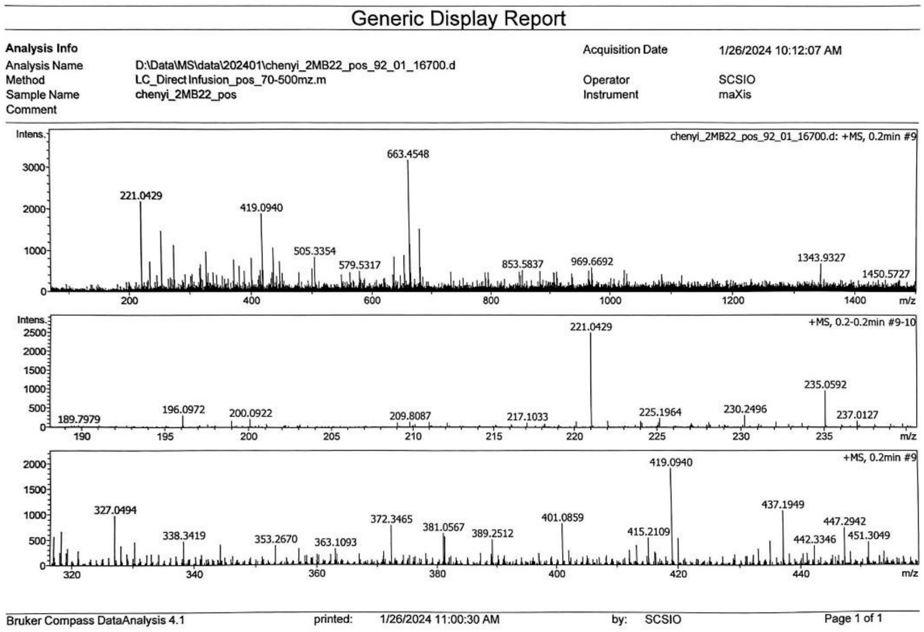

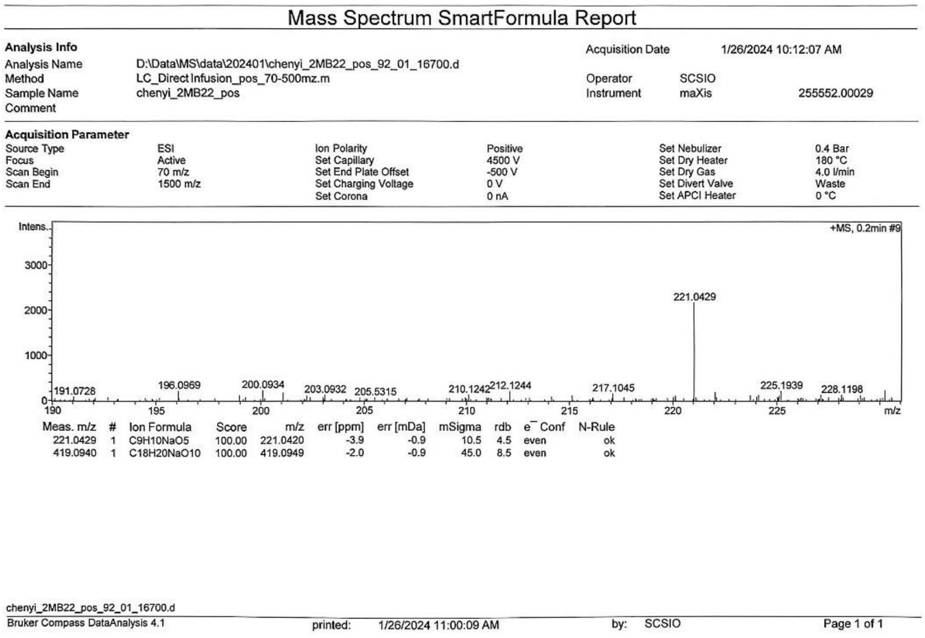

Figure S37: HRESIMS spectrum of **5**.

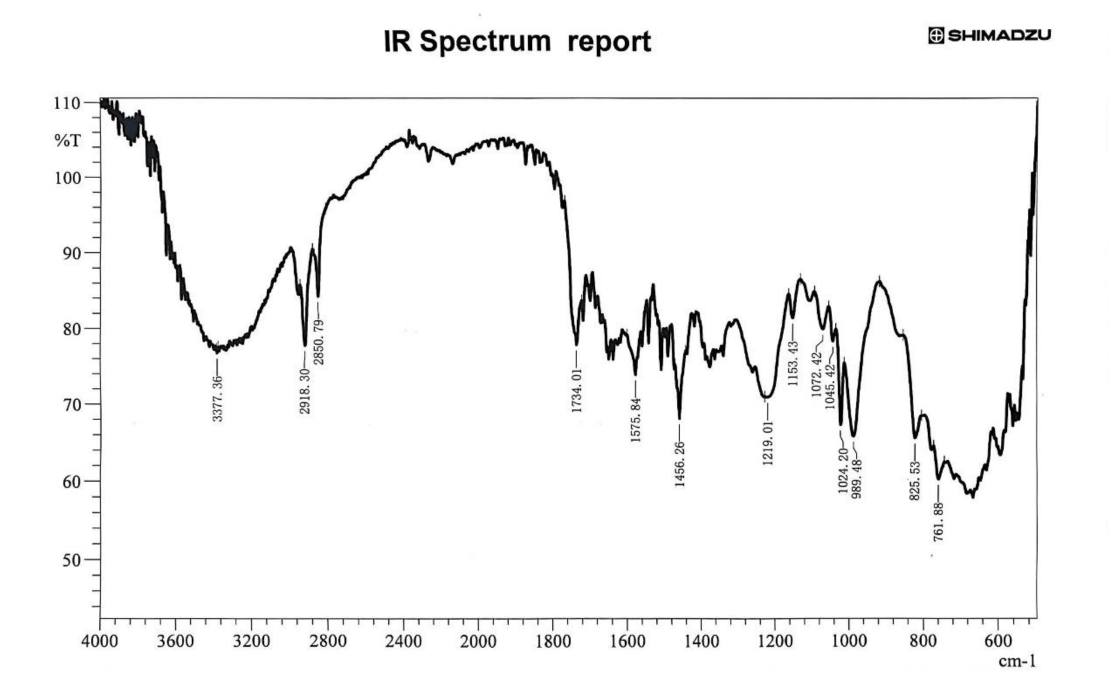

Figure S38: IR spectrum of **5**.

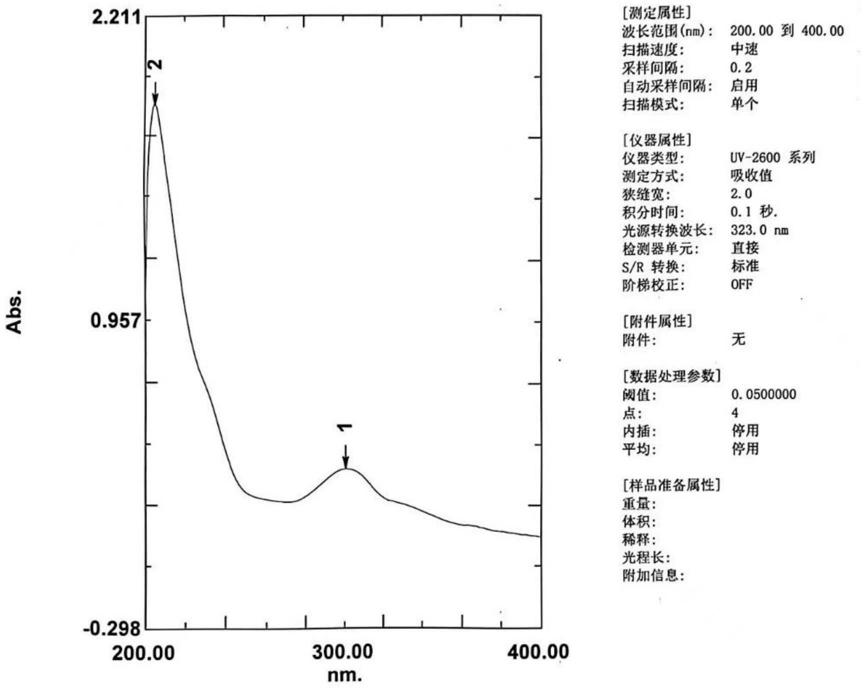

No.	P/V	波长(nm)	Abs.	描述
1	↑	300.60	0.355	
2	↑	205.00	1.841	

Figure S39: UV spectrum of **5** in MeOH.

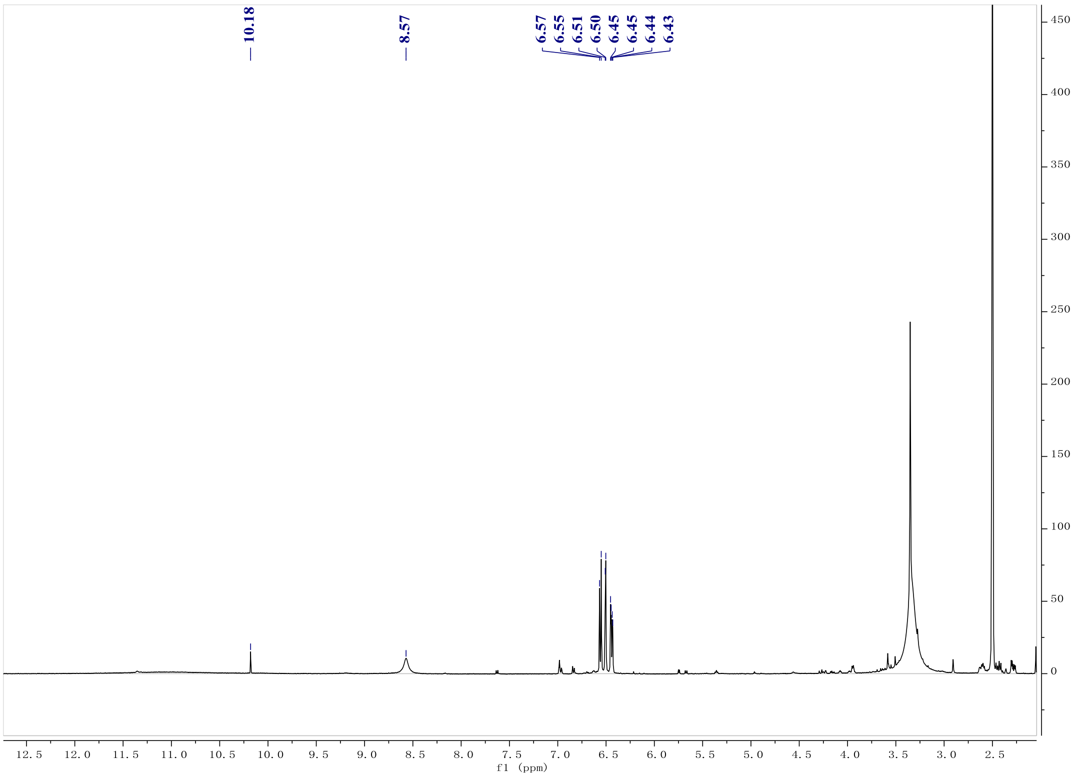

Figure S40: ^{1}H NMR spectrum of **6** in DMSO-d_6.

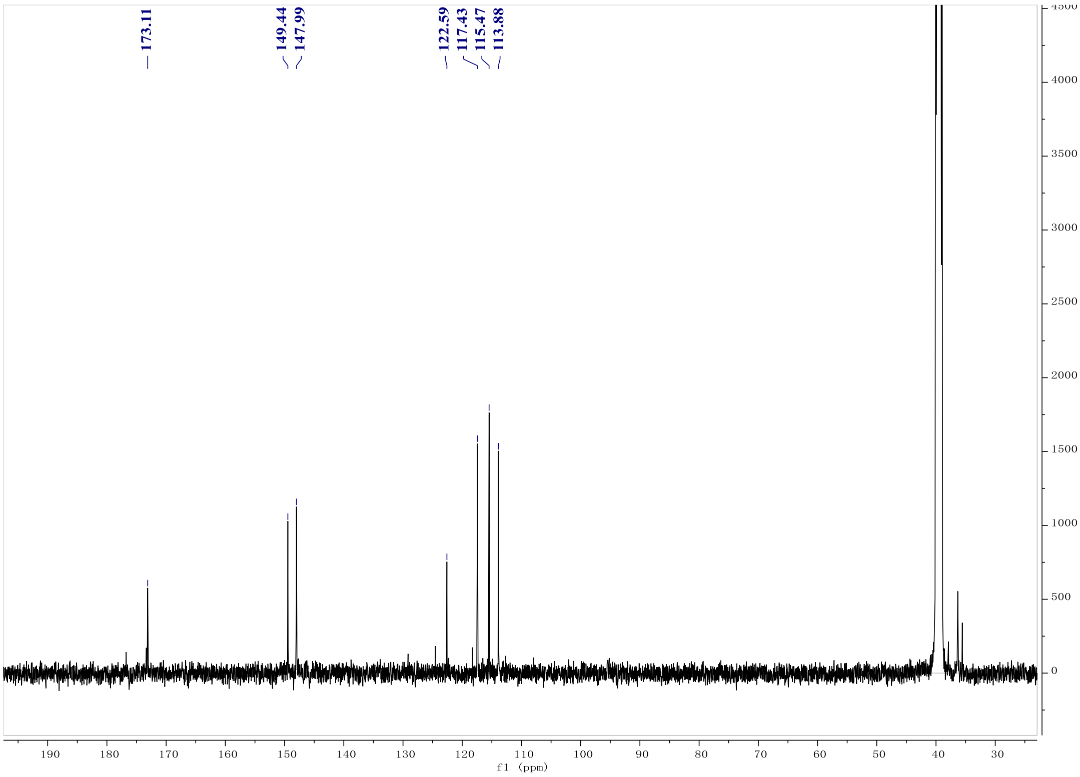

Figure S41: ^{13}C NMR spectrum of **6** in DMSO-d_6.

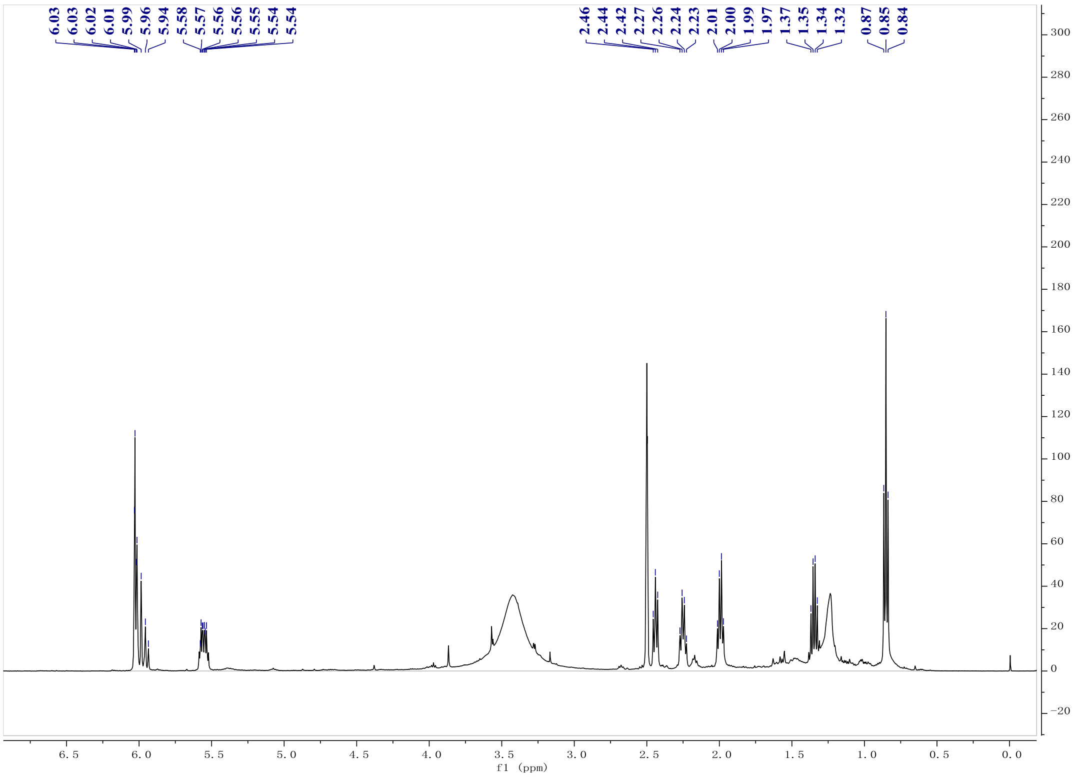

Figure S42: ^{1}H NMR spectrum of **7** in DMSO-d_6.

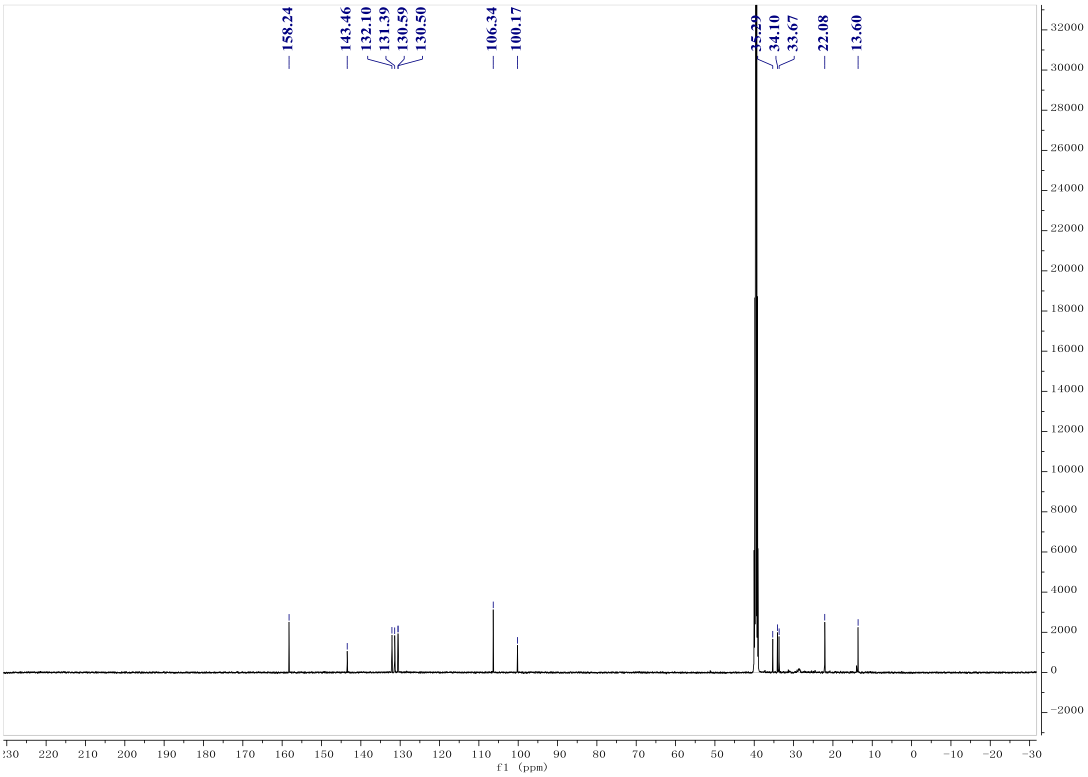

Figure S43: ^{13}C NMR spectrum of **7** in DMSO-d_6.

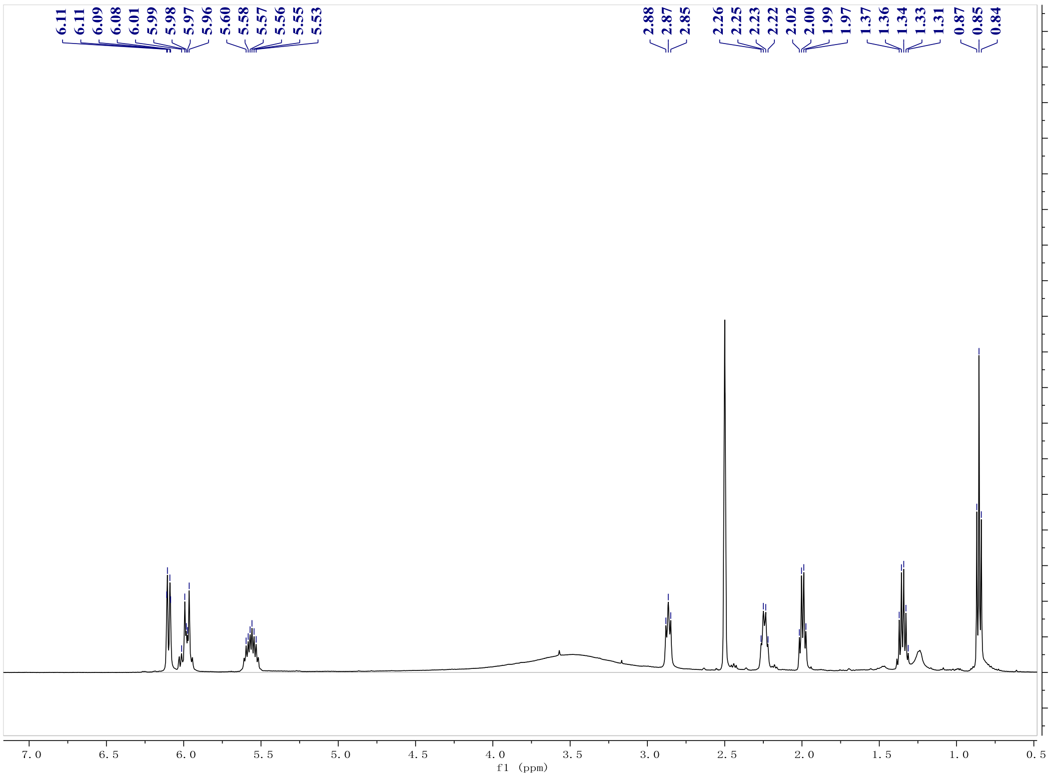

Figure S44: ^{1}H NMR spectrum of **8** in DMSO-d_6.

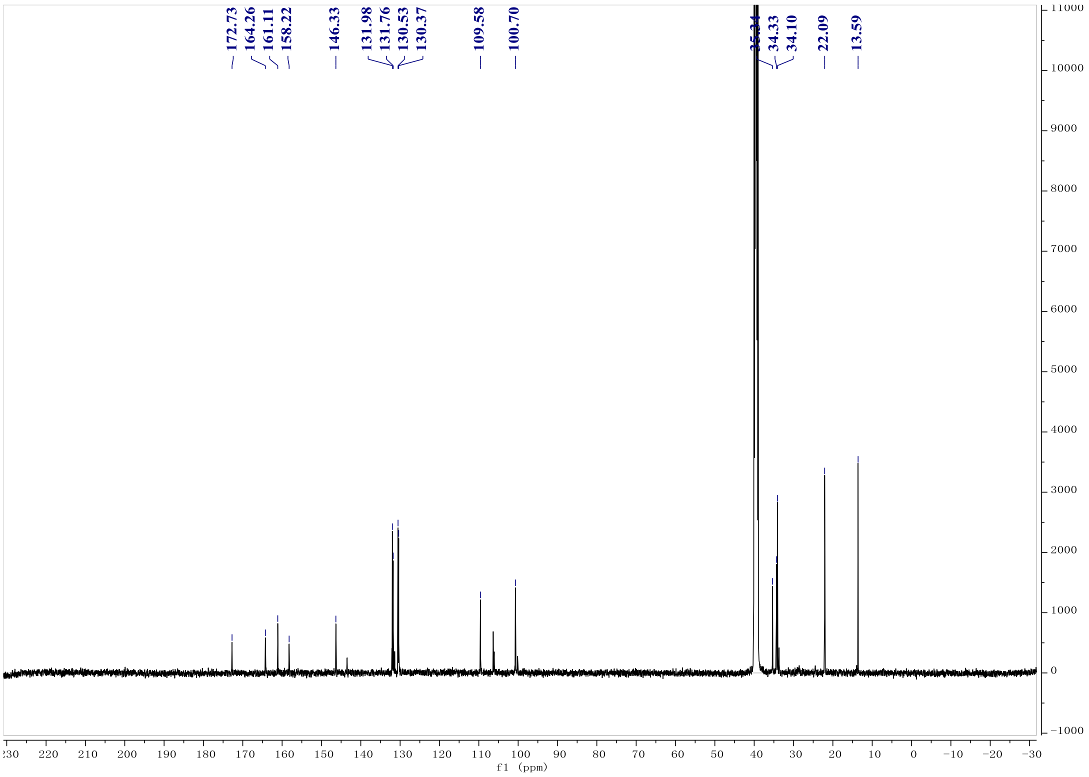

Figure S45: ^{13}C NMR spectrum of **8** in DMSO-d_6.

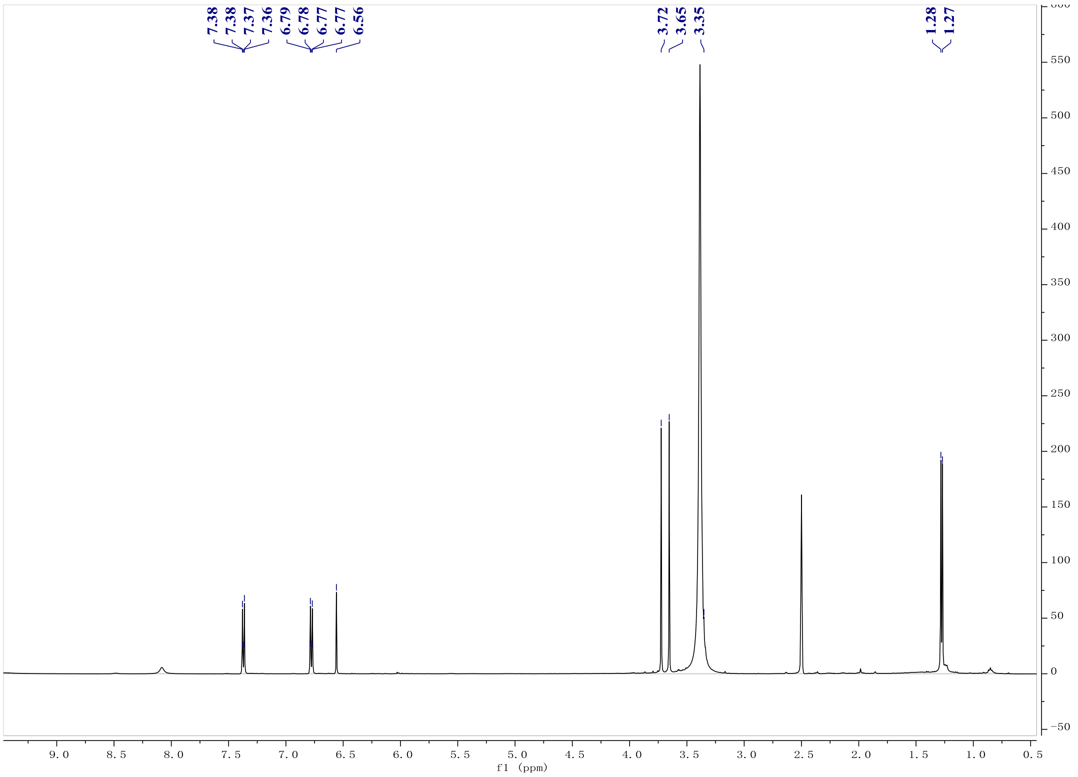

Figure S46: ^{1}H NMR spectrum of **9** in DMSO-d_6.

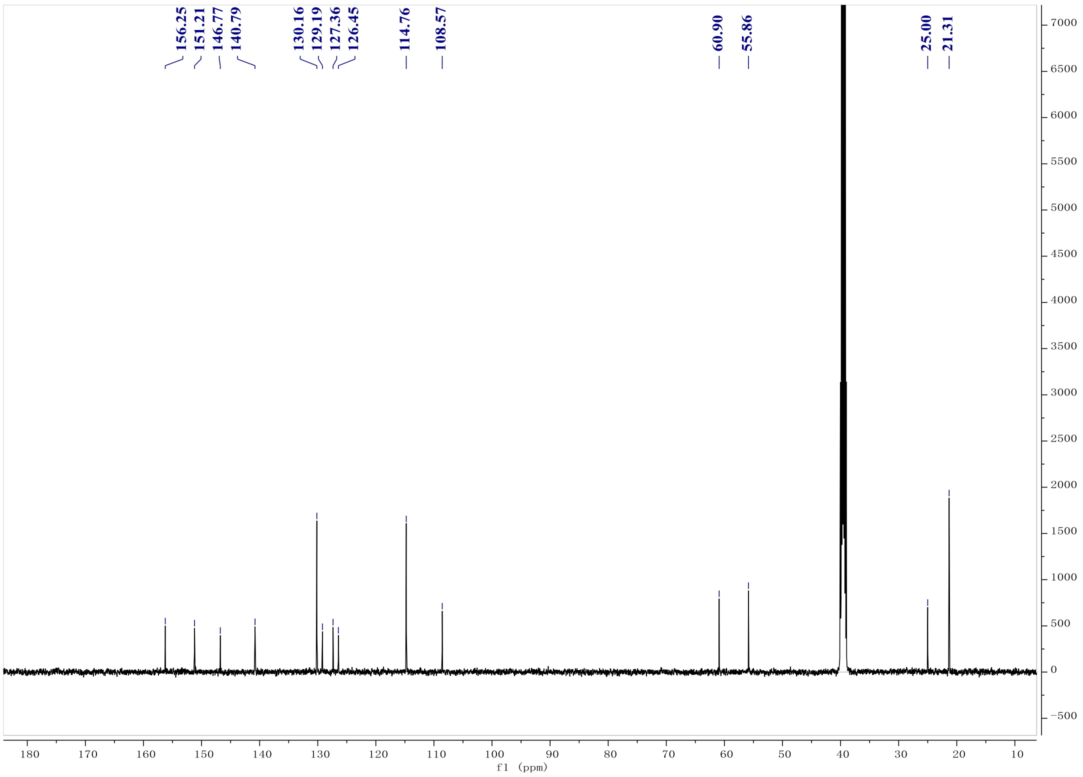

Figure S47: ^{13}C NMR spectrum of **9** in DMSO-d_6.

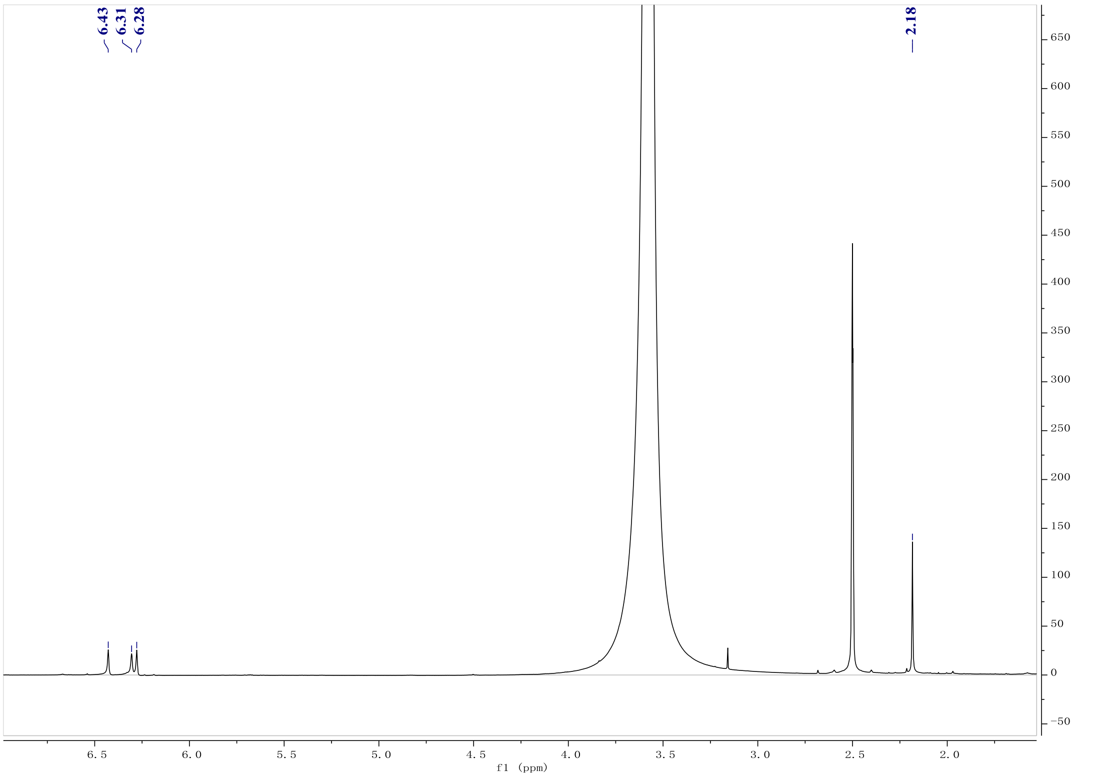

Figure S48: ^{1}H NMR spectrum of **10** in DMSO-d_6.

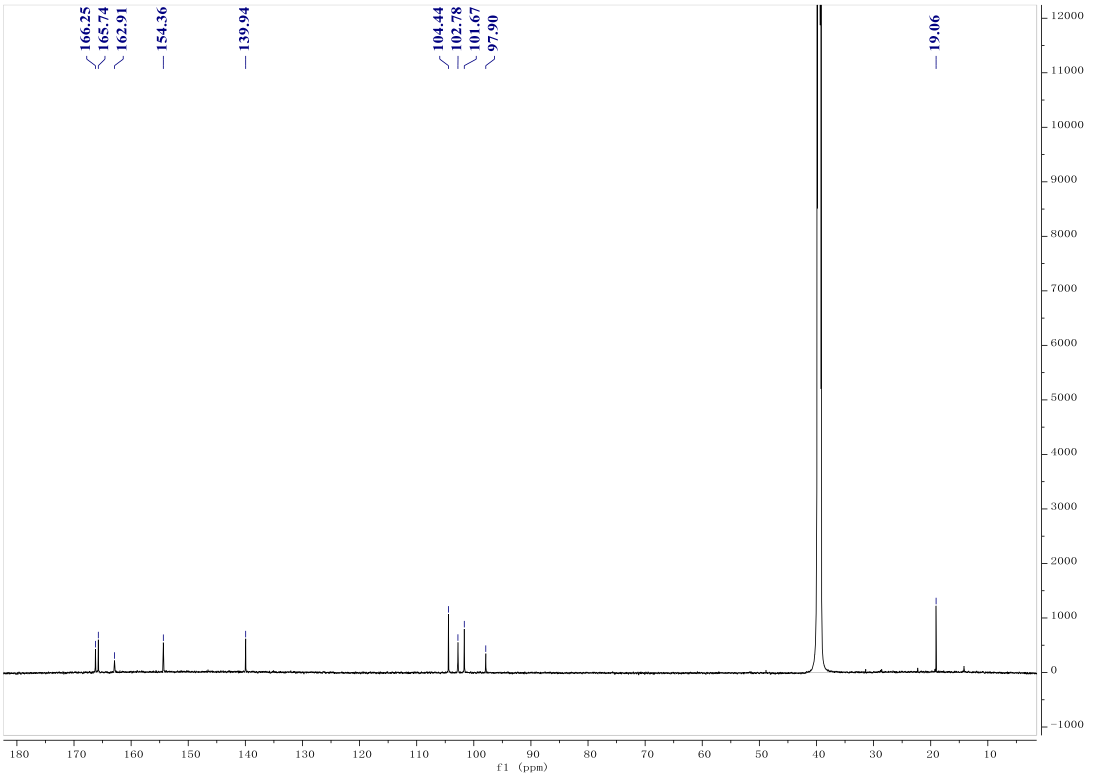

Figure S49: ^{13}C NMR spectrum of **10** in DMSO-d_6.

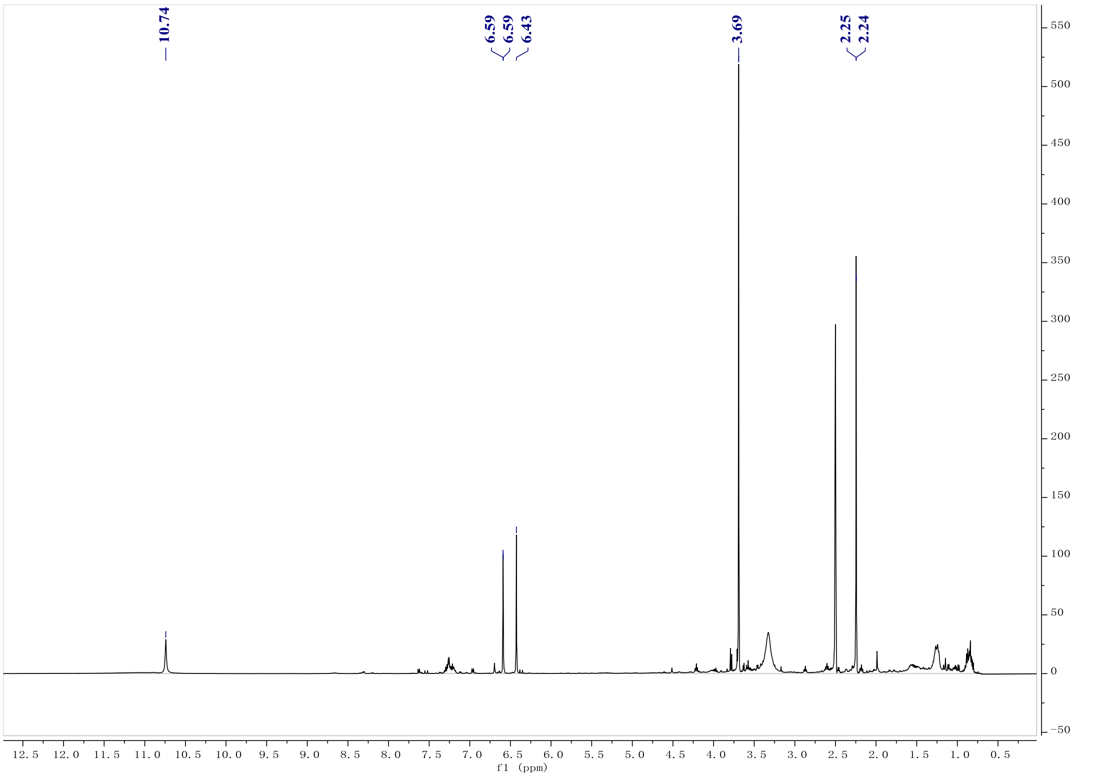

Figure S50: ^{1}H NMR spectrum of **11** in DMSO-d_6.

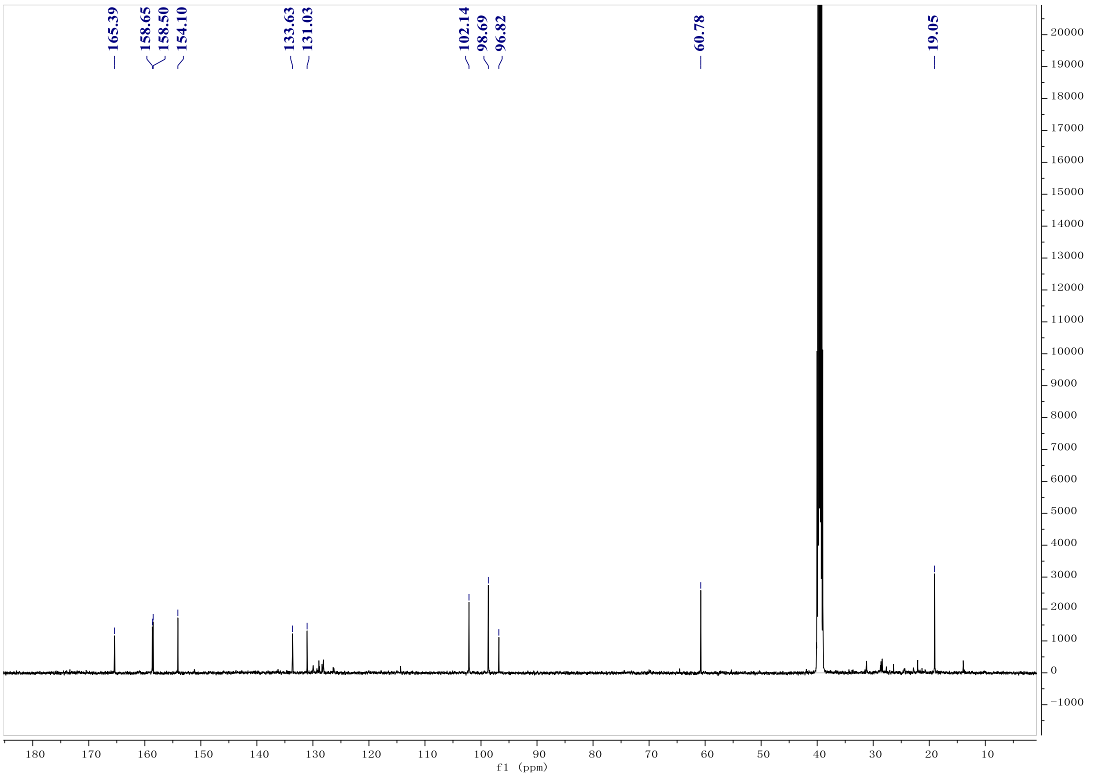

Figure S51: ^{13}C NMR spectrum of **11** in DMSO-d_6.

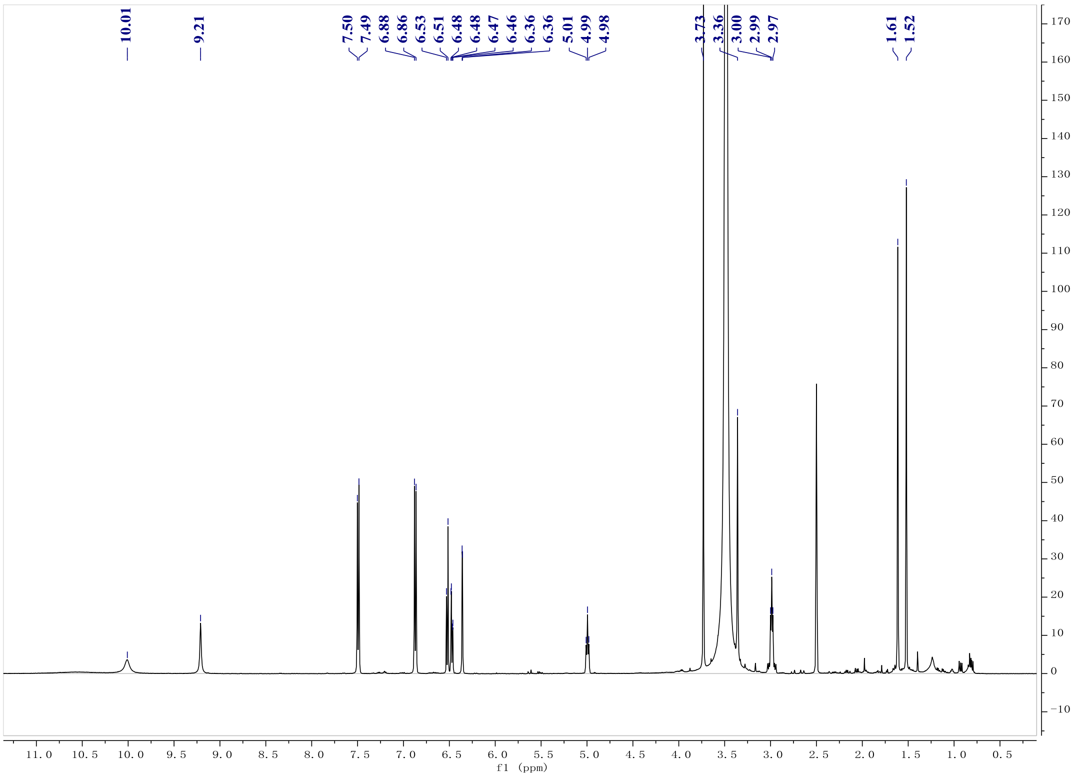

Figure S52: ^{1}H NMR spectrum of **12** in DMSO-d_6.

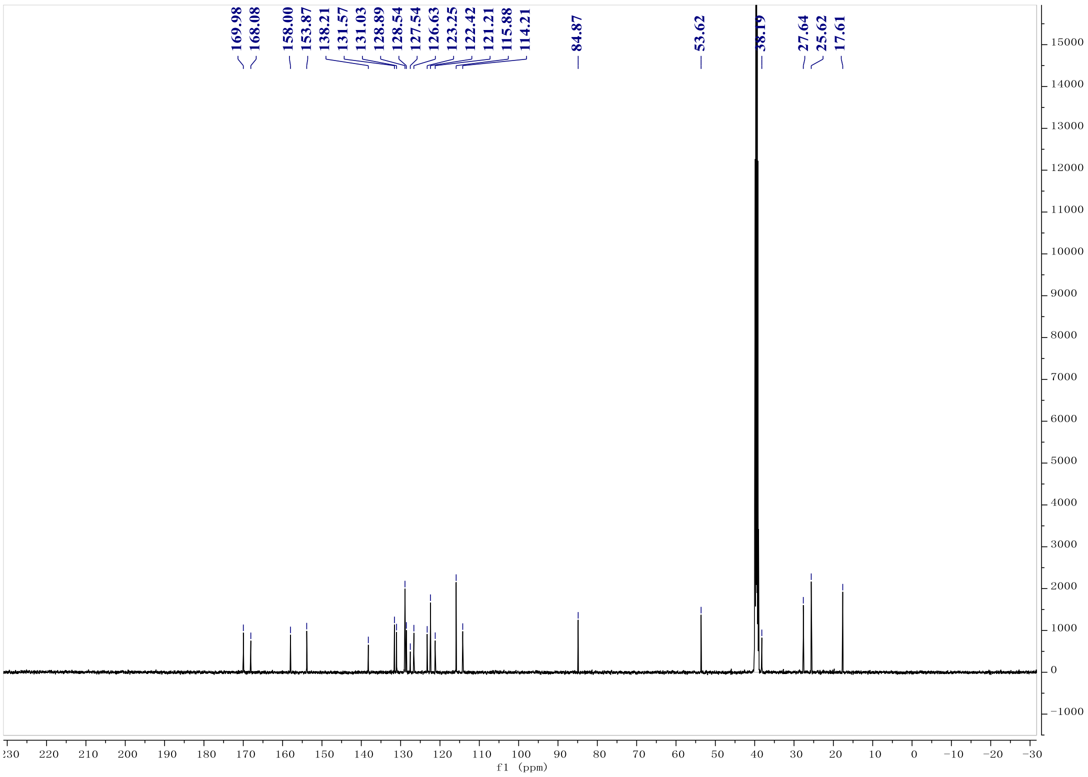

Figure S53: ^{13}C NMR spectrum of **12** in DMSO-d_6.

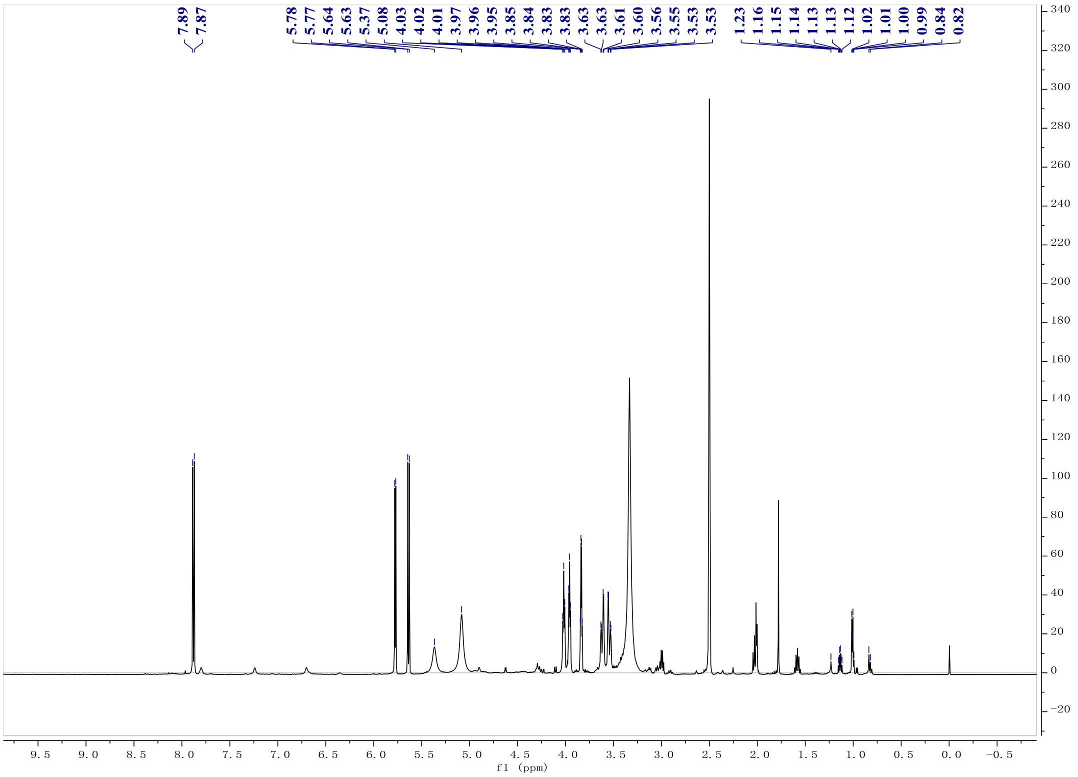

Figure S54: ^{1}H NMR spectrum of **13** in DMSO-d_6.

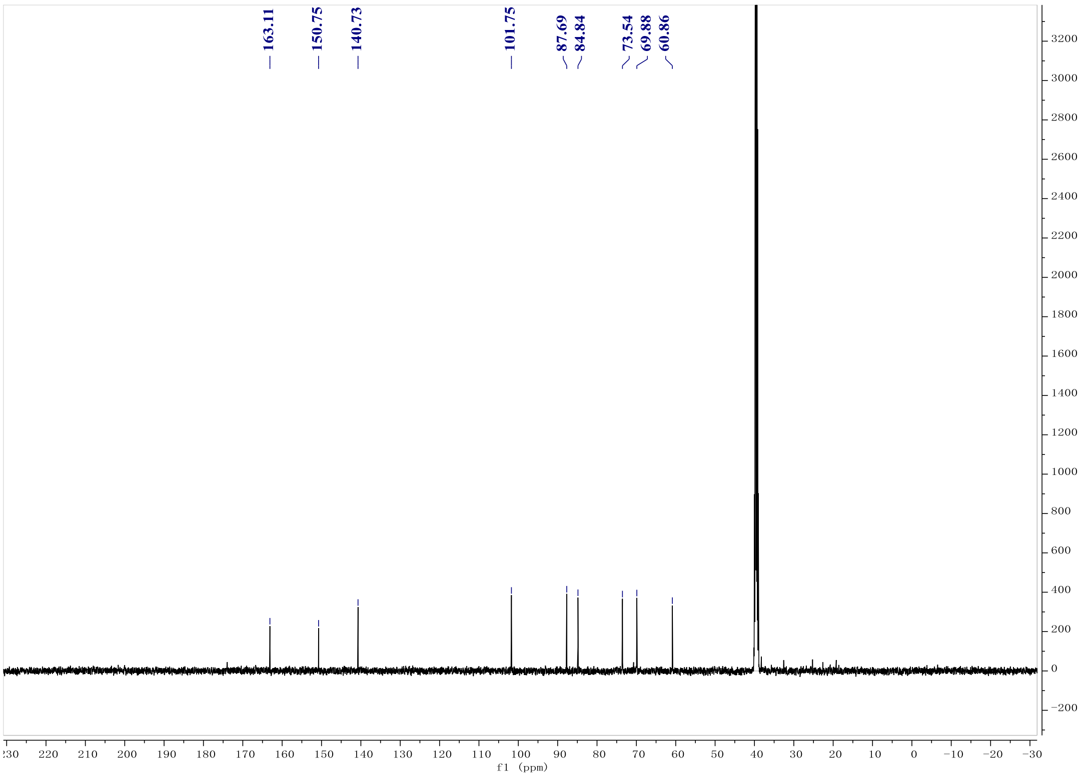

Figure S55: ^{13}C NMR spectrum of **13** in DMSO-d_6.

ITS sequence of the strain *Penicillium* sp. SCSIO 41411

TGATATGCTTAAGTTCAGCGGGTATCCCTACCTGATCCGAGGTCAACCTGAGAAAGATT
GAGGGGGGTCGCCGGCGGGCGCCGGCCGGGCCTACAGAGCGGGTGACGAAGCCCCAT
ACGCTCGAGGACCGGACGCGGTGCCGCCGCTGCCTTTCGGGCCCGCCCCCCGGTAGC
CGGGGGGCGGGGCCCAACACACAAGCCGTGCTTGAGGGCAGCAATGACGCTCGGAC
AGGCATGCCCCCCGGAATACCAGGGGGCGCAATGTGCGTTCAAAGACTCGATGATTCA
CTGAATTCTGCAATTCACATTACTTATCGCATTTCGCTGCGTTCTTCATCGATGCCGGAA
CCAAGAGATCCGTTGTTGAAAGTTTTAACTGATTTAGCTAATCGCTCAGACTGCATTCT
TCAGACAGCGTTCAATGGTGTCTTCGGCGGGCGCGGGCCCGGGGGCGGATGCCCCCC
GGCGGCCGGAACGGCGGGCCCGCCGAAGCAACAAGGTACGATAAACACGGGTGGGA
GGTTGGACCCAGAGGGCCCTCACTCGGAATGATCCTTCCGCA
